# Effect of Red, Processed, and White Meat Consumption on the Risk of Gastric Cancer: An Overall and Dose–Response Meta-Analysis

**DOI:** 10.3390/nu11040826

**Published:** 2019-04-11

**Authors:** Seong Rae Kim, Kyuwoong Kim, Sang Ah Lee, Sung Ok Kwon, Jong-Koo Lee, NaNa Keum, Sang Min Park

**Affiliations:** 1 Department of Medicine, Seoul National University College of Medicine, Seoul 03080, Korea; sungkim20@snu.ac.kr; 2 Department of Biomedical Sciences, Seoul National University Graduate School, Seoul 03080, Korea; kwkim238@gmail.com; 3 Department of Preventive Medicine, College of Medicine, Kangwon National University, Chuncheon-si, Gangwon-do 24341, Korea; sangahlee@kangwon.ac.kr (S.A.L.); kamelon@hanmail.net (S.O.K.); 4 JW Lee Center for Global Medicine, Seoul National University College of Medicine, Seoul 03087, Korea; kcdc7000@gmail.com; 5 Department of Nutrition, Harvard T.H. Chan School of Public Health, Boston, MA 02115, USA; nak212@mail.harvard.edu; 6 Department of Food Science and Biotechnology, Dongguk University, Goyang 10326, Korea; 7 Department of Family Medicine, Seoul National University Hospital, Seoul National University College of Medicine, Seoul 03080, Korea

**Keywords:** white meat, red meat, processed meat, gastric cancer, cancer epidemiology

## Abstract

Whether the risk of gastric cancer varies by the types of meat consumption still remains disputable. The purpose of this meta-analysis was to identify the exact associations that red, processed, and white meat have with gastric cancer. We searched relevant studies in Medline, EMBASE, and the Cochrane Library before November 2018, including cohort and case-control studies. We used random-effect models to estimate the adjusted relative risk (RR), and Egger’s tests to evaluate publication bias. Through stepwise screening, 43 studies were included in this analysis (11 cohort studies and 32 case-control studies with 16,572 cases). In a meta-analysis for the highest versus lowest categories of meat consumption, both red (RR: 1.41, 95% confidence interval (CI): 1.21–1.66) and processed (RR: 1.57, 95% CI: 1.37–1.81) meat consumption were positively associated with gastric cancer risk, while white meat consumption was negatively associated with gastric cancer risk (RR: 0.80, 95% CI: 0.69–0.92). In a dose–response meta-analysis, the RRs of gastric cancer were 1.26 (95% CI: 1.11–1.42) for every 100 g/day increment in red meat consumption, 1.72 (95% CI: 1.36–2.18) for every 50 g/day increment in processed meat consumption, and 0.86 (95% CI: 0.64–1.15) for every 100 g/day increment in white meat consumption. The increase of white meat consumption may reduce the risk of gastric cancer, while red or processed meat may increase the risk of gastric cancer. Further studies are required to identify these associations, especially between white meat and gastric cancer.

## 1. Introduction

For over 50 years, the incidence of gastric cancer has declined worldwide [1]. Gastric cancer, however, remains the fifth most common cancer and the third major cause of cancer death globally [2], which has an enormously negative effect on public health. Therefore, it is important to identify and control the risk factors that make a remarkable contribution to the morbidity and mortality of gastric cancer.

As the etiology of gastric cancer is multifaceted, diverse risk factors concerned with gastric cancer were discovered. Among them, dietary habits have long been considered an important factor in the risk of gastric cancer [3]. Among various dietary habits, meat consumption has steadily increased worldwide in recent years, and red and processed meat, which represent the majority of meat intake, might be a latent risk factor of stomach cancer [4,5].

Nevertheless, the precise contributions of red and processed meat on gastric cancer are still in dispute. The Continuous Update Project (CUP) on gastric cancer which was published in 2016 and based on the stomach cancer section of the Second Expert Report by the World Cancer Research Fund/American Institute for Cancer Research (WCRF/AICR) disclosed that there is strong evidence that high processed meat intake enhances the risk of gastric non-cardia cancer [6]. The CUP, however, which was published this year and based on the Third Expert Report in 2018, changed its judgement on the effect of processed meat consumption on gastric non-cardia cancer from ‘strong evidence’ to ‘limited evidence’ [7]. Furthermore, the associations between red or white meat and gastric cancer still remain unclear because of insufficient research data, and the relation between processed meat intake and gastric cardia cancer also remains inconsistent [7].

Moreover, although previous meta-analyses were carried out to investigate the associations between red or processed meat and stomach cancer [8,9], there has been no meta-analysis to examine evidences on the relationship between white meat and stomach cancer. It is important to investigate the detailed information of white meat as a protein intake source because most past studies mainly focused on the effect of red or processed meat. In addition, there have been no dose–response meta-analysis to examine the associations between processed or white meat intake and gastric cancer risk. Furthermore, there have been no meta-analyses that adjusted *Helicobacter pylori (H. pylori)* infection, which is one of the strongest risk factors for gastric cancer, as a confounder. Therefore, this systematic review and updated meta-analysis was conducted to identify the exact association that red, processed, and white meat have with gastric cancer.

## 2. Materials and Methods

We followed Preferred Reporting Items for Systematic Reviews and Meta-analyses (PRISMA, Table S1) [10] and Meta-analysis of Observational Studies in Epidemiology (MOOSE) guidelines [11] for the design, analysis, and reporting of this meta-analysis.

### 2.1. Literature Search

An online literature search was conducted in MEDLINE (PubMed), EMBASE, and the Cochrane Library until 10 November, 2018, by two independent investigators (SRK and KK). The detailed search terms and words are shown in the Table S2. Based on the search results, the two investigators reviewed the titles, abstracts, and full text, step by step to select relevant studies. Furthermore, backward citation was carried out through the inspection of the references of selected studies to investigate further relevant articles. All the inconsistencies were resolved through discussion between the two investigators.

### 2.2. Study Selection Criteria

Consistent with WCRF/AICR, we defined red meat as beef, pork, lamb, and goat; processed meat as meat preserved by smoking, salting, curing, or meat adding chemical preservatives [12]. Given no definition of white meat by the WCRF/AICR report, we defined white meat as poultry, chicken, duck, turkey, and rabbit based on previous studies’ definition of white meat.

To be included in this meta-analysis, studies had to be case-control or cohort studies that investigated the associations between meat intake (red, processed, or white meat) and gastric cancer risk. Additional inclusion criteria are as follows: (1) articles with information on red, processed, and white meat consumption based on WCRF/AICR criteria, (2) outcome of the study of gastric cancer, (3) reporting odds, hazard ratio (OR, HR) or relative risk (RR) for outcome. If population data were duplicated on several studies, the study with larger sample sizes or longer follow-up period was included in this meta-analysis. Articles were excluded if they met the following exclusion criteria: (1) no data on red, processed, or white meat based on WCRF/AICR criteria, (2) no data on incidence of gastric cancer, (3) letter, review, correspondence or commentary, (4) articles which didn’t include OR, HR, or RR for gastric cancer.

### 2.3. Data Extraction and Quality Assessment

Two investigators (SRK and KK) independently extracted the following information: last name of the author, study year, country, study design, follow-up duration, number of gastric cancer incidences, sample size, type of meat intake, amount of meat consumption of each category including highest and lowest intake, adjusted ORs or RRs with the corresponding 95% confidence interval (CI), and adjustment variables.

Quality evaluation of non-randomized studies, such as cohort studies and case-control studies, is essential for the accurate interpretation of non-randomized studies’ results in a meta-analysis. Low quality studies might distort the results of a meta-analysis. The Newcastle-Ottawa Scale (NOS) is a widely used quality assessment tool, and it assesses the quality of cohort and case-control studies based on their design and contents. The detailed assessment items are suggested in the Tables S3 and S4.

According to the NOS, we evaluated the study quality on a 9-star system [13]. In addition, a study with 7 stars (or more) was defined as a high-quality study on the basis of previous studies which used Newcastle-Ottawa Scale.

### 2.4. Statistical Analysis

Given the generally low incidence of gastric cancer, we assumed that the OR in case-control studies was equal to RR or HR, because OR may approximate the incidence rate ratio (IRR), RR, and HR under the rare disease assumption [14]. We also considered that the differences among the various measures of RR in this study could be disregarded [15]. Therefore, RR was used to estimate the relation between red, processed, white meat and gastric cancer risk. RRs were measured by (1) comparison of the highest consumption group with the lowest consumption group and by a (2) linear dose–response analysis. To calculate a pooled RR and 95% CI, a random effects model was used according to the methodology by DerSimonian and Laird [16]. If RRs were provided discretely according to sex, both RRs were used separately because there was no duplication impact between the two groups.

A dose–response meta-analysis was performed to identify the association among estimates for different dose levels using generalized least-squares trend (GLST) estimation analysis which was established by Greenland and Longnecker [17]. This approach requires the number of cases and person-years or controls for at least three quantitative exposure categories of exposure variables, and requires mean or median intake for categories of consumption levels. The midpoint of the upper and lower bound of each category was allocated as the average consumption if the mean or median intake was not informed. If the lowest category was open-ended, the boundary was assigned as zero. When the highest category was open-ended, the boundary was considered as having same interval length of the preceding category. If the exposures were reported as “servings” or ‘times”, those were transformed into grams using 120 g as a standard portion size for red or white meat and 50 g for processed meat according to WCRF/AICR report [12]. When conducting a dose–response analysis, we used 100 g/day as the increment unit for red or white meat and 50 g/day for processed meat according to the recommended increment units for dose–response meta-analyses by WCRF/AICR report [12]. When the pooled RRs between studies included for a dose–response analysis and those not included for dose–response analysis were compared, there were no significant differences for all kinds of meat consumption (Figures S1, S2, and S3). A non-linear dose–response analysis was not conducted due to the small number of studies.

Subgroup analysis was also executed by the following various factors: study design (cohort and case-control studies, especially population-based and hospital-based), sex, geographic region (Asia, Europe, North America, Latin America, and Oceania), anatomical subtype (cardia and non-cardia), histological subtype (intestinal (differentiated) and diffuse (undifferentiated)), quality score (≥7, high quality study; <7, low quality study), and study adjustment (total energy intake, body mass index (BMI), smoking, alcohol drinking, vegetable intake, fruit intake, salt intake, socioeconomic status (education level, household income, or social class), and *H. pylori* adjustment).

The heterogeneity across studies was evaluated by Q and Higgin’s I^2^ statistics [18]. If the *p* value for heterogeneity was <0.1 or I^2^ was >50%, the studies were considered as heterogeneous. We presented estimates computed by the random effects model for all analyses for consistency regardless of the level of heterogeneity [16].

Publication bias was assessed using Begg’s rank correlation test and Egger’s test [19,20]. As these tests were low powered, we considered that there was evidence of statistical publication bias if the *p* value of two tests was <0.1. Statistical analyses were executed using STATA version 13.0 (Stata Corp., College Station, TX, USA).

## 3. Results

### 3.1. Literature Search and Study Characteristics.

**Figure 1:**
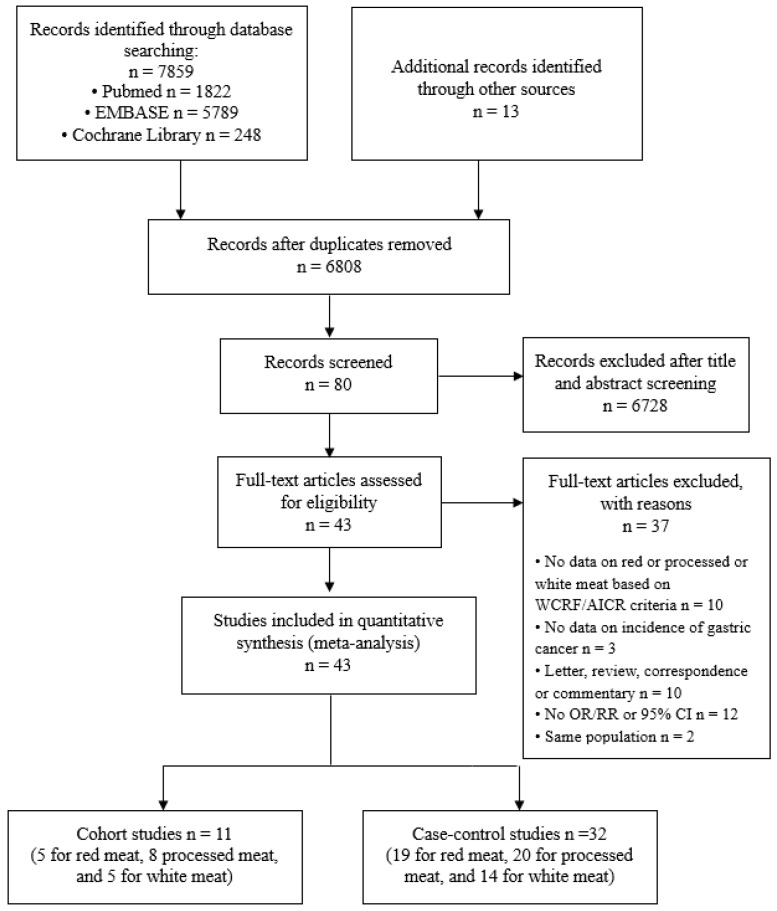
Flow diagram for the selection of studies. A total of 43 studies for quantitative meta-analysis were selected using the Preferred Reporting Items for Systematic Reviews and Meta-analyses (PRISMA) 2009 flow diagram.

Figure 1 shows a PRISMA flow chart of the study selection process [10]. By searching databases (PubMed, EMBASE, and the Cochrane Library) and hand searching relevant bibliographies, 7859 articles were identified. In addition, 13 articles were included from the reference by hand searching. After excluding 1064 duplicated articles, two of investigators independently reviewed and excluded 6728 articles that did not satisfy the predetermined selection criteria based on each article’s title and abstract. The full texts of the 80 remaining articles were reviewed and 37 articles were excluded for the following reasons: 10 articles were removed because they were letter, review, correspondence, or commentary; 12 articles because of no OR/RR or 95% CI; 10 articles because of no data on red, processed, or white meat based on WCRF/AICR criteria; 3 articles because of no data on incidence of gastric cancer; 2 articles because they reported same population. Finally, a total of 43 studies which were published between 1990 and 2017 were included in this meta-analysis. All studies consisted of 11 cohort studies (5 for red meat, 8 for processed meat, and 5 for white meat) [4,21,22,23,24,25,26,27,28,29,30] and 32 case-control studies (19 for red meat, 20 for processed meat, and 14 for white meat) [31,32,33,34,35,36,37,38,39,40,41,42,43,44,45,46,47,48,49,50,51,52,53,54,55,56,57,58,59,60,61,62]

Table 1 illustrates the general characteristics of the studies in this meta-analysis. A total of 1,764,894 subjects and 4314 stomach cancer patients in 11 cohort studies were included in this study. In addition, a total of 76,806 controls and 12,258 cases in the 32 case-control studies were included in this analysis. A total of 16 studies were performed in Asia, 12 in Europe, 9 in North America, 5 in Latin America, and 1 in Oceania. Six studies conducted their analysis according to the subtypes of gastric cancer (cardia and non-cardia). Adjustment confounders were various among the studies, such as total energy intake, BMI, smoking habits, drinking, vegetable intake, fruit intake, salt intake, and socioeconomic status, but most studies adjusted age and sex as covariates. The quality assessment score of the studies is summarized in the Tables S3 and S4. The score ranged from 4 to 9 stars on the scale. The cohort studies’ median score was 9, and case-control studies’ median score was 7.

### 3.2. Red Meat and Gastric Cancer

#### 3.2.1. Highest Versus Lowest Consumption

**Figure 2:**
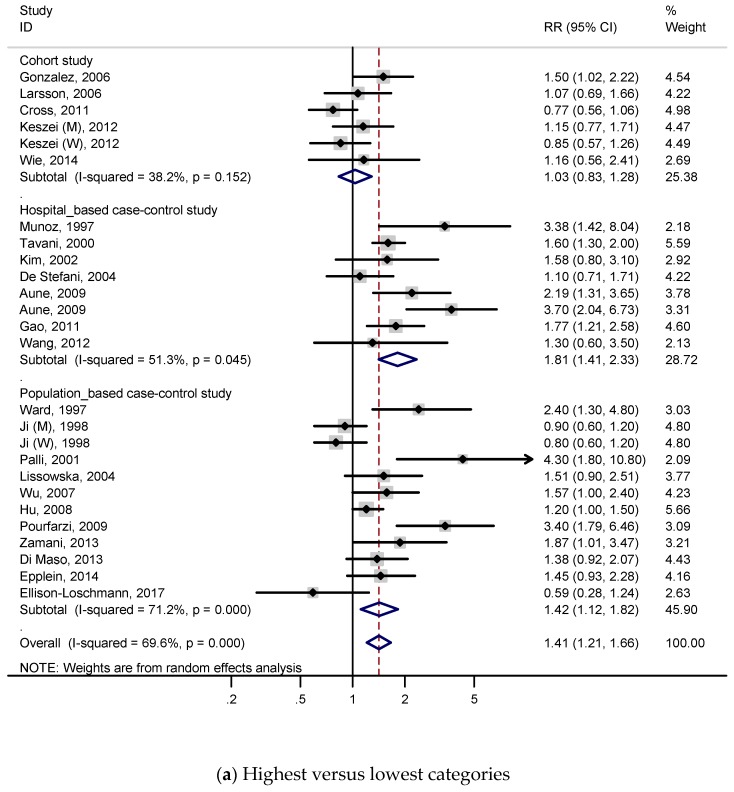

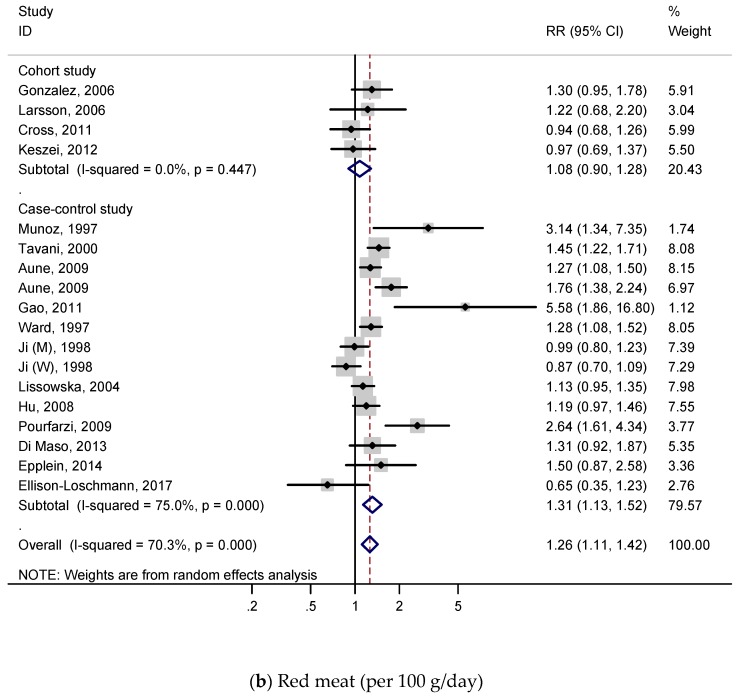
Adjusted relative risks and 95% confidence intervals of gastric cancer for red meat consumption. Squares indicate each study’s relative risks. Horizontal lines indicate 95% confidence intervals. Diamonds indicate the summary relative risks and 95% confidence intervals. (**a**) Highest versus lowest categories of red meat consumption; (**b**) dose–response meta-analysis for 100 g/day increase in red meat consumption.

A total of 5 cohort studies and 19 case-control studies with 9726 cases were included to identify the association between red meat intake and the risk of gastric cancer. Figure 2 presents the adjusted RRs for each study and pooled RRs for relevant or all studies. RRs were calculated by comparing the highest red meat consumption with the lowest red meat consumption (Figure 2a). As shown in Figure 2a, the subjects with a higher intake of red meat had a 41% higher risk of gastric cancer in the meta-analysis (RR, 1.41; 95% CI, 1.21–1.66), and we found statistically significant heterogeneity (*p* < 0.001; I^2^, 69.6%). According to the Figure S4a, publication bias was discovered on Begg’s test (*p* = 0.022) and Egger’s test (*p* = 0.076).

A subgroup analysis for red meat was conducted to investigate study heterogeneity, and the results are presented in Table 2. When conducting a subgroup analysis according to a study design, significant relations were detected for case-control studies (total RR 1.57, 95% CI 1.30–1.89; population-based RR 1.42, 95% CI 1.12–1.82; hospital-based RR 1.81, 95% CI 1.41–2.33), but these were not detected for cohort studies (RR 1.03, 95% CI 0.83–1.28). There were no significant associations when the subgroup analyses were conducted according to sex, anatomical subtype, or histological subtype. In an analysis by geographic region, a positive association was observed on Asian (RR, 1.40; 95% CI, 1.04–1.89), European (RR, 1.48; 95% CI, 1.15–1.92), and Latin American populations (RR, 2.03; 95% CI, 1.01–4.06), but this was not observed on the North American population. In an analysis by quality score, significant association was found when studies were limited to a quality score ≥7 (RR, 1.43; 95% CI, 1.21–1.68). Statistically positive associations were detected for all adjustment models: when studies were adjusted for total energy intake (RR, 1.37; 95% CI, 1.14–1.64), BMI (RR, 1.23; 95% CI, 1.01–1.50), smoking (RR, 1.34; 95% CI, 1.11–1.61), alcohol drinking (RR, 1.23; 95% CI, 1.01–1.49), vegetable intake (RR, 1.35; 95% CI, 1.10–1.66), fruit intake (RR, 1.43; 95% CI, 1.16–1.78), salt intake (RR, 3.40; 95% CI, 1.79–6.46), socioeconomic status (RR, 1.40; 95% CI, 1.17–1.68), and *H. pylori* (RR, 1.88; 95% CI, 1.04–3.40).

#### 3.2.2. Dose–Response Analysis

A total of 4 cohort studies and 13 case-control studies were included for the dose–response meta-analysis of red meat consumption and gastric cancer risk. As shown in Figure 2b, the subjects had a 26% higher risk of gastric cancer per 100 g/day increment in red meat consumption (RR, 1.26; 95% CI, 1.11–1.42), and we found statistically significant heterogeneity (*p* < 0.001; I^2^, 70.3%). According to Figure S4b, publication bias was not discovered on Begg’s test (*p* = 0.472) and Egger’s test (*p* = 0.315).

Subgroup analysis for red meat was performed to investigate study heterogeneity, and the results are presented on Table 3. As shown in Table 3, the overall trends of most subgroup analysis were consistent with those of the subgroup meta-analysis for highest versus lowest red meat consumption categories.

### 3.3. Processed Meat and Gastric Cancer

#### 3.3.1. Highest Versus Lowest Consumption

**Figure 3:**
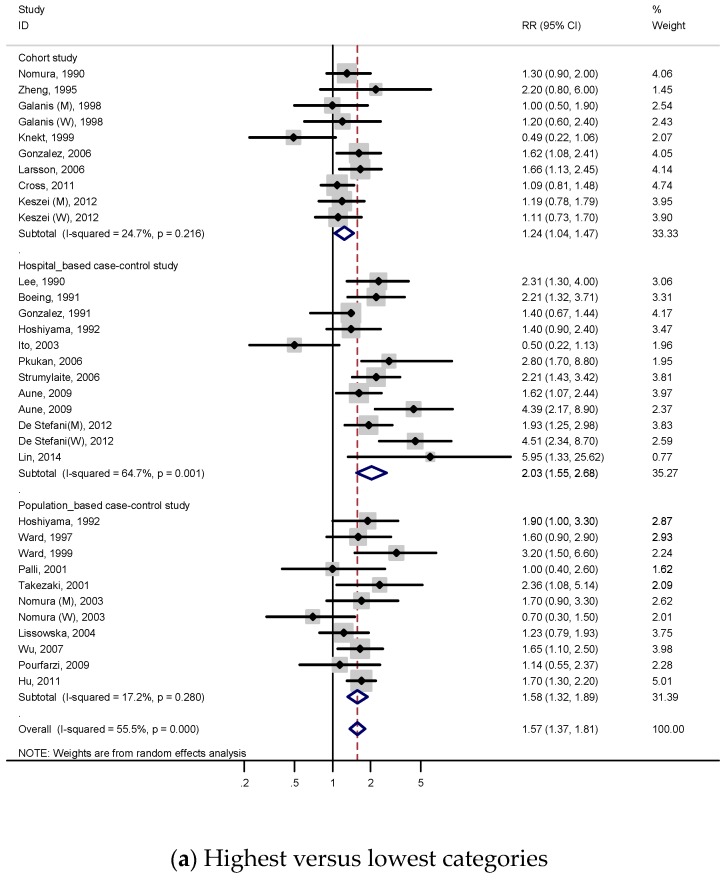

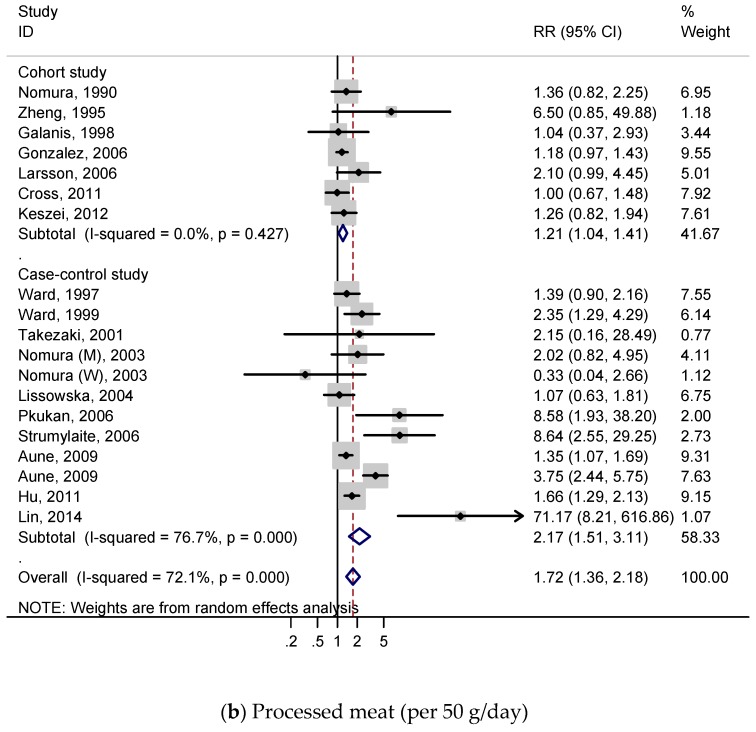
Adjusted relative risks and 95% confidence intervals of gastric cancer for processed meat consumption. Squares indicate each study’s relative risks. Horizontal lines indicate 95% confidence intervals. Diamonds indicate the summary relative risks and 95% confidence intervals. (**a**) Highest versus lowest categories of processed meat consumption; (**b**) dose–response meta-analysis for 50 g/day increase in processed meat consumption.

A total of 8 cohort studies and 20 case-control studies with 10,645 cases were involved to examine the association between processed meat consumption and gastric cancer. According to Figure 3a, the subjects with a higher consumption of processed meat had a 57% higher stomach cancer risk (RR, 1.57; 95% CI, 1.37–1.81), and significant heterogeneity was discovered (*p* < 0.001; I^2^, 55.5%). As shown in Figure S5a, publication bias was not detected on both Begg’s test (*p* = 0.556) and Egger’s test (*p* = 0.448).

Table 2 presents the results of the subgroup analysis for processed meat intake. In most subgroup models, trends and results were consistent with the total summary results. Positive associations were observed on cohort studies (RR 1.24; 95% CI, 1.04–1.47), case-control studies (total RR 1.79, 95% CI 1.51–2.12; population-based (RR 1.58, 95% CI 1.32–1.89); hospital-based (RR 2.03, 95% CI 1.55–2.68), and men group (RR 1.40; 95% CI, 1.13–1.74), but these were not observed in the women group. In an analysis by geographic region, significant relations were detected on the populations of all regions (Asian populations RR 1.74, 95% CI 1.22–2.48; European populations RR 1.40, 95% CI 1.14–1.73; North American populations RR 1.36, 95% CI 1.15–1.61; Latin American populations RR 2.69, 95% CI 1.76–4.12). In terms of anatomical subtype, significant association was detected on the non-cardia group (RR, 1.34; 95% CI, 1.10–1.63), but was not observed on the cardia group. When stratified by histological subtype, no significant associations were detected on histological subtype groups. In analysis by quality score, positive relations were found on a quality score ≥7 group (RR, 1.50; 95% CI, 1.28–1.75) and quality score <7 group (RR, 1.96; 95% CI, 1.55–2.49). Significantly positive associations were observed on almost all adjustments models except for salt intake and the *H. pylori* infection model (total energy intake RR 1.47, 95% CI 1.20–1.80; BMI RR 1.89, 95% CI 1.51–2.36; smoking RR 1.68, 95% CI 1.37–2.07; alcohol drinking RR 2.34, 95% CI 1.81–3.02; vegetable intake RR 2.02, 95% CI 1.60–2.56; fruit intake RR 1.72, 95% CI 1.42–2.10; socioeconomic status RR 1.57, 95% CI 1.33–1.85).

#### 3.3.2. Dose–Response Analysis

A total of 7 cohort studies and 11 case-control studies were included for the dose–response meta-analysis of processed meat consumption and gastric cancer risk. As shown in Figure 3b, the subjects had a 72% higher risk of gastric cancer per 50 g/day increment in processed meat consumption (RR, 1.72; 95% CI, 1.36–2.18), and we found statistically significant heterogeneity (*p* < 0.001; I^2^, 72.1%). According to Figure S5b, publication bias was discovered on Begg’s test (*p* = 0.086) and Egger’s test (*p* = 0.039).

A subgroup analysis for processed meat was conducted to investigate study heterogeneity, and the results are presented on Table 3. Table 3 shows that the overall trends of most subgroup analyses was similar with those of the subgroup meta-analysis for highest versus lowest processed meat consumption categories.

### 3.4. White Meat and Gastric Cancer

#### 3.4.1. Highest Versus Lowest Consumption

**Figure 4:**
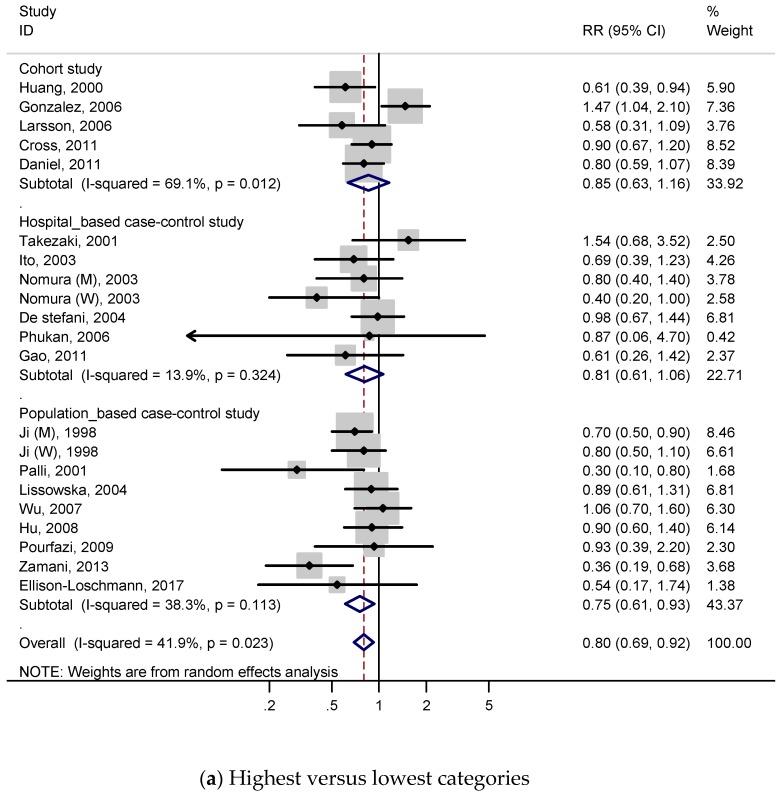

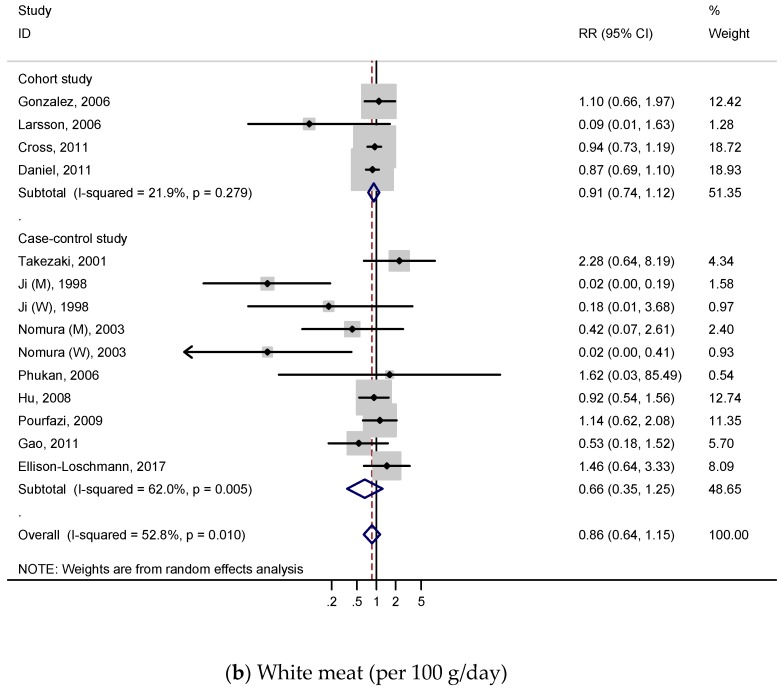
Adjusted relative risks and 95% confidence intervals of gastric cancer for white meat consumption. Squares indicate each study’s relative risks. Horizontal lines indicate 95% confidence intervals. Diamonds indicate the summary relative risks and 95% confidence intervals. (**a**) Highest versus lowest categories of white meat consumption; (**b**) dose–response meta-analysis for 100 g/day increase in white meat consumption.

A total of 5 cohort studies and 14 case-control studies with 9896 cases were included to investigate the relationship between white meat intake and gastric cancer, and RRs were reported in Figure 4a. For all studies, the summary RR indicated a 20% decreased risk of gastric cancer (RR, 0.80; 95% CI, 0.69–0.92), which contrasted with the result of red meat or processed meat. We identified low heterogeneity (*p* = 0.023; I^2^, 41.9%) and no publication bias both on Begg’s test (*p* = 0.131) and Egger’s test (*p* = 0.116), according to Figure S6a.

In the subgroup analysis (Table 2), a negative association was detected when the analysis was restricted to study quality score ≥7 (high-quality) (RR, 0.80; 95% CI, 0.68–0.93) and case-control studies (RR, 0.77; 95% CI, 0.66–0.91). On the other hand, there was no significant association in cohort studies group (RR, 0.85; 95% CI, 0.63–1.16) and quality score <7 (low-quality) (RR, 0.80; 95% CI, 0.54–1.18). When stratified by sex, a negative relation was observed on women group (RR, 0.67; 95% CI, 0.52–0.87). In the subgroup analysis by geographic region, negative association was only detected on Asian populations (RR, 0.70; 95% CI, 0.57–0.85), and was not detected on European, North American, and Latin American populations. Negative association was also observed in studies adjusted for total energy intake (RR, 0.80; 95% CI, 0.64–1.00) and socioeconomic status (RR, 0.80; 95% CI, 0.67–0.96), but not observed in other adjustments, which were BMI, smoking, alcohol drinking, vegetable intake, fruit intake, salt intake, and *H. pylori*.

#### 3.4.2. Dose–Response Analysis

Four cohort studies and eight case-control studies were included for the dose–response meta-analysis of white meat consumption and gastric cancer risk. As shown in Figure 4b, the effect reducing gastric cancer risk was slightly attenuated. The pooled RR of gastric cancer per 100 g/day increment in white meat consumption was 0.86 (95% CI 0.64–1.15), and we found statistically significant heterogeneity (*p* = 0.010; I^2^, 52.8%). According to Figure S6b, publication bias was not discovered on Begg’s test (*p* = 0.208), but was detected on Egger’s test (*p* = 0.096).

Subgroup analyses for white meat were performed to investigate study heterogeneity, and the results are presented on Table 3. The overall trends of most subgroup analyses were similar with those of the subgroup meta-analysis for highest versus lowest red meat consumption categories, but those were attenuated.

## 4. Discussion

In a comprehensive and updated meta-analysis, we found that high consumption of red and processed meat was significantly associated with an increased risk of gastric cancer (41% and 57% increased risk, respectively), while that of white meat was significantly associated with a decreased risk of gastric cancer (20% decreased risk). These results and trends were consistent with those of dose–response meta-analyses (26% increased risk for every 100 g/day increase consumption in red meat and 72% increased risk for every 50 g/day increase consumption in processed meat). Generally, significant associations were observed on case-control studies, and these were also detected on cohort studies in processed meat. In all kinds of meats, positive associations were also observed on Asian populations and quality score ≥7 groups.

There have been various studies to identify the underlying biological mechanism for the association between red and processed meat consumption and gastric cancer. Among gastric cancer risk factors, heme iron, which is abundantly contained in red meat promotes endogenous formation of carcinogenic N-nitroso compounds (NOCs) [63]. Since NOCs affect the high degree of nitrogenous remnants in the gastrointestinal tract, which contributes to formation of DNA adducts, they are considered as risk factors of gastric cancer, especially non-cardia gastric cancer [64,65,66,67]. Furthermore, iron inducing DNA damage or oxidative stress is a critical factor for bacterial growth in *H. pylori*, which is a serious leading risk factor for gastric cancer [68,69].

In addition, for processed meat, the cooking method or processing and storage methods may independently increase the risk of gastric cancer. That is, the cooking method or type of processing and preserving of the meat may also be one of the risk factors for gastric cancer. For example, heterocyclic amines and polycyclic aromatic hydrocarbons are produced when meat is cooked at high temperature [70]. In addition, high dietary salt, which is contained in cured or salted meat hurts gastric mucosa and induces significant gastric pathology and inflammation [71].

Interestingly, a higher white meat intake was negatively associated with gastric cancer risk. In terms of the fundamental biological mechanism of the association between white meat intake and gastric cancer, lesser heme iron in white meat may hinder an increase in the risk of gastric cancer because it contributes to the suppression of an increase of endogenous formation of NOCs [63,72]. In addition, white meat is an abundant source of polyunsaturated fatty acids (PUFAs) and contains a lower level of cholesterol and saturated fat than red meat [73]. PUFAs are considered to obstruct carcinogenesis by inducing apoptosis, controlling cell cycle and eicosanoid production, and causing anti-proliferative action [74]. Especially, poultry meat which represents the majority of white meat, is a rich provider of omega-3 (n-3) PUFAs [75]. N-3 PUFA inhibits IL-1 and TNF synthesis which are pro-inflammatory cytokines [76]. Thus, it may contribute to reducing the risk of gastric cancer because chronic inflammation is an important factor for inducing carcinogenesis. In addition, n-3 PUFAs seem to block β-catenin and cyclooxygenase-2 (COX-2) in other organ’s carcinoma cells, which also might contribute to the prevention of gastric cancer [77].

Westerners (Europeans or Americans) consume a larger amount of white meat quantitatively than Asians, and also white meat consumption constitutes a larger proportion of total meat consumption in Westerners than that in Asians [78]. Considering that the incidence rate of gastric cancer in Asians was much higher than that in Westerners, a larger amount of white meat consumption might contribute to reducing gastric cancer risk in addition to the harmful effects of the traditional Asian foods which have a tendency to be salted [79]. Of course, we cannot discard residual confounding factors, as a high intake of white meat might indicate overall healthier eating patterns [27]. In addition, since the effect of reducing gastric cancer risk was attenuated when conducting dose–response analysis, further prospective studies should be performed to identify the exact association between white meat intake and gastric cancer risk.

When stratified by study design, stronger associations between meat consumption and the risk of gastric cancer were observed in case-control studies compared with cohort studies on all kinds of meat. These differences may in part result from the recall bias because case-control studies depend on the patient’s memory of the past. Furthermore, selection bias also may have contributed to the discrepancy between two kinds of studies because the RR was especially higher in hospital-based case-control studies. When classified by geographic region, the relative risks were different depending on geographic regions in all kinds of meats. These different risk values reflect the possibility that regional eating habits may affect the risk of gastric cancer. Especially, regarding only Asian populations with the highest white meat intake had a 30% lower risk of gastric cancer in our study, therefore further investigation is required to identify the associations among ethnicity which is related to geographic region, eating patterns, white meat consumption, and the risk of gastric cancer. When stratified by anatomical type, the increase of risk of gastric cancer was observed on the non-cardia cancer group in processed meat. On the other hand, there were no significant associations between other kinds of meats intake and anatomical subtype (cardia and non-cardia cancer). In addition, no significant associations between all kinds of meat consumption and histological subtype (intestinal (differentiated) and diffuse (undifferentiated)) were observed. Thus, more evidential data are required to identify the influence of meat intake to anatomical subtype or histological subtype of gastric cancer. In the subgroup analysis according to adjustments, significantly high RRs were observed on red meat intake regardless of all potential confounding factors (total energy intake, BMI, smoking, alcohol drinking, vegetable intake, fruit intake, salt intake, socioeconomic status, and *H. pylori* infection). Interestingly, when adjusted for *H. pylori*, red meat intake was still significantly related to gastric cancer risk. Regarding this result, we considered that a high level of red meat consumption may increase the risk of gastric cancer regardless of the *H. pylori* infection, which is one of the strongest risk confounders for gastric cancer. We also considered that red or processed meat consumption independently contributes to increasing the risk of gastric cancer, regardless of various important confounders.

This meta-analysis study has several limitations. First, although various confounding factors were adjusted, some residual confounders may not be excluded because each study had different adjustments. Although many studies were adjusted for total energy, BMI, smoking, alcohol drinking, vegetable intake, fruit intake, and socioeconomic status, some critical confounders, such as *H. pylori* infection, were still not adjusted. We obtained significant results only on red meat after adjusting *H. pylori* infection. To identify the accurate associations between processed, white meat, and gastric cancer, more studies, which are adjusted for *H. pylori* infection or other important confounders, should be performed. Second, significantly stronger associations between meat intake and the risk of gastric cancer were detected in case-control studies compared with cohort studies. Generally, cohort studies are more reliable and robust than case-control studies because cohort studies are comparatively free from recall bias or selection bias. In prospective cohort studies, exposure data are collected before the subject’s pathogenesis and are collected from participants who share identical characteristics except for their exposure status of interest. Therefore, further prospective cohort studies are required to identify the exact associations. Third, the results in this study might be slightly influenced by the difference of the definition of meat category because each study had a different criteria of meat categorization. For example, some studies defined processed red meat as ‘red meat’, but it can be also included in ‘processed meat’. Finally, although publication bias was not the problem for processed and white meat, it might have affected the result for red meat. Heterogeneity across studies was also detected in some subgroup analyses, which might have overstated the association between meat consumption and the risk of gastric cancer. However, the publication bias was not detected on the dose–response analysis for red meat, and results were similar with the highest versus lowest consumption analysis.

Despite the limitations, our study is highly important because it was the first meta-analysis study to identify the association that white meat intake has with gastric cancer. In addition, this is the first study to identify the dose–response associations between processed or white meat intake and gastric cancer risk. Moreover, our study is more reliable because this is the updated meta-analysis study reflecting recent researches about the association between red or processed meat consumption and gastric cancer after previous meta-analyses. Furthermore, this is the first meta-analysis to investigate the results on studies adjusted for *H. pylori* infection, which is one of the strongest risk factors of gastric cancer. The results in our meta-analysis represented the worldwide large-scale population because 1,764,894 participants and 4314 gastric cancer patients were involved in cohort studies including 76,806 controls and 12,258 cases in case-control studies. These abundant data provided more reliable and robust results, and the analysis in this study was statistically more reliable thanks to the huge scale of population data. Regarding the confined evidences concerning white meat consumption and the risk of gastric cancer [80], future studies are required to identify the association between white meat intake and gastric cancer. Through these prospective studies, exact influences of white meat intake on gastric cancer might be discovered.

In conclusion, the increased consumption of red and processed meat is positively related to the risk of gastric cancer. On the other hand, white meat intake seems to have a negative association with the risk of gastric cancer. It is a very important issue because red and processed meat consumption still accounts for the largest portion of overall meat intake in developed countries including the United States [5]. Our findings suggest that white meat intake might be associated with reduction of the risk of gastric cancer, and white meat can be considered as a great source of protein. Further studies should be conducted in well-designed cohort studies or clinical trials to identify these associations accurately.

## Figures and Tables

**Table 1:** Characteristics of included studies associated with red, processed, and white meat consumption and gastric cancer risk.

Study	Study Type	No. of Cases	No. of Controls or Cohort Size	Study Period (Follow-Up Duration, Year)	Type of Meat	Consumption Comparison Category	Adjusted ORs/RRs (95% CI)	Adjusted Variables
Nomura et al. (1990) USA [21]	CO	150	7990	1965–1968 (19 years)	Processed meat (ham, bacon, and sausage)	≥5 vs. <1 (times/week)	1.3 (0.9–2.0)	Age
Zheng et al. (1995) USA [30]	CO	26	34,691	1986–1992	Processed meat	≥13 vs. <4.4 (times/month)	2.20 (0.8–6.0)	Age, education, smoking status, and pack-years of smoking
Galanis et al. (1998) USA [22]	CO	108	11,907	1975–1980 (14.8 years)	Processed meat	≥3 vs. =0 (times/week)	1.0 (0.6–1.7)	Age, sex, education, and Japanese place of birth
Knekt et al. (1999) Finland [23]	CO	68	9989	1966–1972 (24 years)	Processed meat (cured meat)	Q4 vs. Q1 (quartiles)	0.49 (0.22–1.06)	Age, sex, municipality, smoking, and energy intake
Huang et al. (2000) Japan [24]	CO	877	1386	1988–1994 (11 years)	White meat (chicken)	≥3–4 vs. =0 (times/week)	0.61 (0.39–0.94)	Age, gender, and pathological type and stage of cancer.
Gonzalez et al. (2006) Europe [4]	CO	348	521,457	1992–1998 (6.5 years)	Red, processed, white meat (poultry)	Red meat (g/day)	1.5 (1.02–2.22)	Sex, height, weight, education, alcohol use, smoking, physical activity, energy intake, fruit and vegetable intake, and other meats intake.
						(Man) ≥84 vs. <26		
						(Woman) ≥61 vs. <17		
						Processed meat (g/day)	1.62 (1.08–2.41)	
						(Man) ≥59 vs. <16		
						(Woman) ≥37 vs. <9		
						White meat (g/day)	1.47 (1.04–2.10)	
						(Man) ≥29 vs. <7		
						(Woman) ≥26 vs. <5		
Larsson et al. (2006) Sweden [25]	CO	156	61,433	1987–1997 (18 years)	Red meat (beef, pork, lamb, or veal), processed meat (bacon, side pork, sausage, hot dogs, ham, or salami), and white meat (poultry)	Red meat (times/week)	1.07 (0.69–1.66)	Age, education, BMI, energy intake, alcohol, fruits and vegetables intake
						≥3.5 vs. <2		
						Processed meat (times/week)	1.66 (1.13–2.45)	
						≥3 vs. <1.5		
						White meat (times/week)	0.58 (0.31–1.09)	
						≥0.5 vs. <0.2		
Cross et al. (2011) USA [26]	CO	955 (454 cardia and 501 non-cardia)	494,979	1996–2006 (10 years)	Red, processed, and white meat	Red meat (grams/1000 kcal)		Age, education, sex, BMI, ethnicity, smoking, alcohol drinking, physical activity, daily intake of fruits, vegetables, saturated fat, and calories
						(Cardia) ≥64.8 vs. <10.0	1.04 (0.72–1.51)	
						(Non-cardia) ≥64.8 vs. <10.0	0.77 (0.56–1.06)	
						Processed meat (g/1000 kcal)		
						(Cardia) ≥23.2 vs. <1.7	0.82 (0.59–1.14)	
						(Non-cardia) ≥23.2 vs. <1.7	1.09 (0.81–1.48)	
						White meat (g/1000 kcal)		
						(Cardia) ≥65.8 vs. <9.7	1.18 (0.87–1.60)	
						(Non-cardia) ≥65.8 vs. <9.7	0.90 (0.67–1.20)	
Daniel et al. (2011) USA [27]	CO	928 (418 cardia and 510 non-cardia)	492,186	1995–2006 (9 years)	White meat (poultry)	(Cardia) ≥51.2 vs. <5.3 (grams/1000 kcal)	1.00 (0.73–1.36)	Red meat intake, age, sex, education, marital status, family history of cancer, race, body mass index, smoking status, frequency of vigorous physical activity, menopausal hormone therapy in women, intake of alcohol, fruit, and vegetables, fish intake, and total energy
						(Non-cardia) ≥51.2 vs. <5.3 (grams/1000 kcal)	0.80 (0.59–1.07)	
Keszei et al. (2012) Netherlands [28]	CO	652 (men, 139 cardia and 329 non-cardia; women, 24 cardia and 160 non-cardia)	120,852	1986–2002 (16.3 years)	Red meat (beef, pork, minced meat, liver, and other non-poultry meat) and processed meat (sausage, bacon, ham, cold cuts, croquettes, and frankfurters)	Red meat (g/day)		Age, smoking, energy intake, BMI, alcohol intake, vegetable intake, fruit intake, education and non-occupational physical activity
						men		
						(Cardia) ≥145.9 vs. <45.8	1.00 (0.56–1.78)	
						(Non-cardia) ≥145.9 vs. <45.8	1.15 (0.77–1.71)	
						women		
						(Cardia) ≥115.9 vs. <46.9	0.45 (0.16–1.19)	
						(Non-cardia) ≥115.9 vs <46.9	0.85 (0.57–1.26)	
						Processed meat (g/day) men		
						(Cardia) ≥45.5 vs <3.7	1.49 (0.81–2.75)	
						(Non-cardia) ≥45.5 vs <3.7	1.19 (0.78–1.79)	
						women		
						(Cardia) ≥26.0 vs. <3.5	1.12 (0.36–3.47)	
						(Non-cardia) ≥26.0 vs. <3.5	1.11 (0.73–1.70)	
Wie et al. (2014) Korea [29]	CO	46	8024	2004–2013 (7 years)	Red meat	≥43 vs. <43 (g/day)	1.16 (0.56–2.41)	Age, sex, energy intake, BMI, physical activity, smoking, alcohol use, income, education, and marital status.
Lee et al. (1990) Taiwan [31]	CC	210	820	.	Processed meat (cured meat)	≥2 vs. <1 (meals/month)	2.31 (1.3–4.0)	Age, sex, and hospital
Boeing et al. (1991) Germany [32]	CC	143	579	1985–1988	Processed meat	T3 vs. T1 (tertiles)	2.21 (1.32–3.71)	Age, sex, hospital, and intake of raw vegetables, citrus fruits, cheese, and wholemeal bread
González et al. (1991) Spain [33]	CC	354	354	1987–1989	Processed meat (cured meat)	≥57 vs. <3 (g/day)	1.4 (0.8–2.2)	Intakes of preserved fish, egg, cooked vegetables, other fruits, nuts, dried fruits, meat, and total calories
Hoshiyama et al. (1992) Japan [34]	CC	294 population-based	294 population-based	1984–1990	Processed meat (smoked food, bacon, ham)	≥2 vs. none (times/week)	Hospital-based: 1.9 (1.0–3.3)	Age, sex, area, and smoking status
		202 hospital-based	294 hospital-based				Population-based: 1.4 (0.9–2.4)	
Munoz et al. (1997) Italy [35]	CC	88	103	1985–1992	Red meat	≥5 vs. ≤2 (times/week)	3.38(1.42–8.04)	Sex, age, area of residence, and education
Ward et al. (1997) USA [36]	CC	176	449	1988–1993	Red meat (beef, processed meats, fresh ham/pork, and liver) and processed meat (bacon, sausage, processed ham, home-cured meats, and sandwich meats)	Red meat (times/week)	2.4 (1.3–4.8)	Sex, age
						>19 vs. <8		
						Processed meat (times/week)	1.6 (0.9–2.9)	
						>8 vs. <4		
Ji et al. (1998) China [37]	CC	1124 (770 men, 353 women)	1451 (819 men, 632 women)	1988–1989	Red meat (pork chops, pork spareribs, pig feet, fresh pork, beef, and mutton) and white meat (poultry, chicken, duck)	Red meat (times/month) ≥30.7 vs. ≤8.5	Red meat	Age, income, education, smoking (males only). and alcohol drinking (males only)
							(men) 0.9 (0.6–1.2)	
							(women) 0.8 (0.6–1.2)	
						white meat (times/month) ≥2.5 vs. ≤0.7	White meat	
							(men) 0.7 (0.5–0.9)	
							(women) 0.8 (0.5–1.1)	
Ward et al. (1999) Mexico [38]	CC	220	752	1989–1990	Processed meat	≥6 vs. <1 (times/week)	3.2 (1.5–6.6)	Age, sex, total calories, chili pepper consumption, added salt, history of peptic ulcer, cigarette smoking, and socioeconomic status
Tavani et al. (2000) Italy [39]	CC	745	7990	1983–1996	Red meat	≥6 vs ≤3 (portions/week) an average Italian portion is 100 to 150 g	1.6(1.3–2.0)	Age, year of recruitment, sex, education, smoking habits and alcohol, fat, fruit and vegetable intakes
Palli et al. (2001) Italy [40]	CC	382	561	1985–1987	Red meat (beef, pork, lamb, and game), processed meat (cured and canned meats), and white meat (poultry and rabbit)	Red, processed, and white meat	Red meat	Age, sex, social class, family history of GC, area of residence, BMI tertiles, total energy, and consumption tertiles of each food of interest
							(MSI+) 4.3 (1.8–10.8)	
							(MSI−) 2.1 (1.2–3.7)	
						T3 vs. T1 (tertiles)	Processed meat	
							(MSI+) 1.0 (0.4–2.6)	
							(MSI−) 1.9 (1.0–3.7)	
							White meat	
							(MSI+) 0.3 (0.1–0.8)	
							(MSI−) 0.9 (0.5–1.6)	
Takezaki et al. (2001) China [41]	CC	187	333	1996–2000	Processed meat (salted meat) and white meat (poultry)	Processed meat (times/month)	2.36 (1.08–5.14)	Age, sex, and smoking and drinking habits.
						≥4 vs. <1		
						White meat (times/month)	1.54 (0.68–3.52)	
						≥12 vs. <1		
Kim et al. (2002) Korea [42]	CC	136	136	1997–1998	Red meat (grilled beef and pork over charcoal)	Q4 vs. Q1 (quartiles)	1.58 (0.80–3.10)	Sex, age, socioeconomic status, family history and refrigerator use
Ito et al. (2003) Japan [43]	CC	508	36,490	1988–1999	Processed and white meat (chicken)	Processed and white meat	0.50 (0.22–1.13)	Age, year, season at first hospital visit, smoking habits, and family history of gastric cancer
						≥5 vs <1 (times/week)	0.69 (0.39–1.23)	
Nomura et al. (2003) USA [44]	CC	300 (186 men, 114 women)	446 (282 men, 164 women)	1993–1999	Processed and white meat (poultry)	Processed meat (g/day)	Processed meat	Age, ethnicity, cigarette smoking status, education, history of gastric ulcer, NSAID use, family history of gastric cancer, total calories, and intake of other foods or food groups
						(men) >27.2 vs. <9.2	(men) 1.7 (0.9–3.3)	
						(women) >14.6 vs. <6.1	(women) 0.7 (0.3–1.5)	
						White meat (g/day)	White meat	
						(men) >26.5 vs. <12.8	(men) 0.8 (0.4–1.4)	
						(women) >20.3 vs. <11.2	women) 0.4 (0.2–1.0)	
De stefani et al. (2004) Uruguay [45]	CC	240	960	1996–2000	Red meat (beef and lamb), processed meat (salted meat), and white meat (poultry, fish)	Red, processed, and white meat	1.10 (0.71–1.71)	Age, sex, residence, urban/rural status, education, body mass index, and total energy intake
							1.98 (1.35–2.90)	
						T3 vs T1 (tertiles)	0.98 (0.67–1.44)	
Lissowska et al. (2004) Poland [46]	CC	274	463	1994–1996	Red meat (pork, beef, liver, and processed red meats), processed meat (Sausage and hot dog), and white meat (poultry)	Red meat (times/week)	1.51 (0.90–2.51)	Age, sex, education, smoking, and calories from food
						>14.5 vs. <8		
						Processed meat (times/week)	1.23 (0.79–1.93)	
						>4.9 vs. <2.1		
						White meat (times/week)	0.89 (0.61–1.31)	
						≥0.7 vs. <0.7		
Phukan et al. (2006) India [47]	CC	329	658	2001–2004	Processed meat (Smoked dried salted meat) and white meat (chicken)	Processed and white meat (times/week)	2.8 (1.7–8.8)	Level of education, tobacco use, alcohol drinking, and each dietary variable
						≥2 vs. none	0.87 (0.06–4.70)	
Strumylaite et al. (2006) Lithuania [48]	CC	379	1139	2002–2004	Processed meat (salted meat)	≥1–2 vs. Almost do not use (times/week)	2.21 (1.43–3.42)	Smoking, alcohol consumption, family history of cancer, body mass index, education level, residence, diet (salt preserved food items, bread, noodles, rice, different dairy products, mayonnaise, eggs, carrots, cabbage, broccoli, tomatoes, garlic, onion, paprika, bean, potatoes), and physical activity
Wu et al. (2007) USA [49]	CC	623	1308	1992–1997	Red, processed, and white meat (poultry)	Red, processed, and white meat	(Cardia)	Age, sex, race, birthplace, education, smoking, BMI, reflux, use of vitamins, and total calories
							1.56 (0.97–2.5)	
							0.76 (0.5–1.2)	
							1.16 (0.8–1.8)	
						Q4 vs. Q1 (quartiles)	(Non-cardia)	
							1.57 (1.0–2.4)	
							1.65 (1.1–2.5)	
							1.06 (0.7–1.6)	
Hu et al. (2008) Canada [50]	CC	1182	5039	1994–1997	Red, processed, and white meat (poultry)	Red meat (times/week)	1.2 (1.0–1.5)	Age, province, education, body mass index, sex, alcohol use, pack-year smoking, total vegetable and fruit intake, and total energy intake
						≥5.1 vs. ≤2		
						Processed meat (times/week)	1.7 (1.3–2.2)	
						≥5.42 vs. ≤0.94		
						White meat (oz/week)	0.9 (0.6–1.4)	
						≥13 vs. ≤4		
Aune et al. (2009) Uruguay [51]	CC	275	2032	1996–2004	Red meat (fresh beef and lamb) and processed meat	Red meat (g/day)	2.19 (1.31–3.65)	Age, sex, residence, education, income, interviewer, smoking status, cigarettes per day, duration of smoking, age at starting, years since quitting, alcohol, dairy foods, grains, fatty foods, fruits and vegetables, fish, poultry, mate drinking, BMI and energy intake; red meat was adjusted for processed meat and vice versa
						250–600 vs. <150		
						Processed meat (g/day)	1.62 (1.07–2.44)	
						40–258.8 vs. <10		
Aune et al. (2009) Uruguay [52]	CC	128	1832	1988–2000	Red and processed meat	Red meat (servings/week)	3.70 (2.04–6.73)	Age, sex (when applicable), education, residence, smoking status, cigarettes per day, age at starting smoking, years since quitting smoking, duration of smoking, type of tobacco, alcohol intake, fruits and vegetables and milk.
						≥9 vs. ≤4		
						Processed meat (servings/month)	4.39 (2.17–8.90)	
						>1 vs. 0		
Pourfarzi et al. (2009) Iran [53]	CC	213	390	2003–2005	Red, processed, and white meat (chicken)	Red meat (times/week)	3.40 (1.79–6.46)	Sex, age group, education, family history of GC, citrus fruits, garlic, onion, red meat, fish, dairy products, strength and warmth of tea, preference for salt intake, and *H. pylori*
						>7 vs. ≤2		
						Processed meat (times/week)	1.14 (0.55–2.37)	
						≥0.25 vs. never		
						White meat (times/week)	0.93 (0.39–2.20)	
						≥7 vs. ≤2		
Gao et al. (2011) China [54]	CC	915	1514		Red and white meat (chicken)	Red meat	(Cardia)	Age, gender, geographic region
						>weekly vs. monthly/seldom/never	1.54 (1.15–2.07) (Non-cardia) 1.77 (1.21–2.58)	
						White meat	(Cardia)	
							0.98 (0.52-1.86) (Non-cardia)	
						daily/weekly vs. never	0.61 (0.26–1.42)	
Hu et al. (2011) Canada [55]	CC	1182	5039	1994–1997	Processed meat (hot dogs, luncheon meat, smoked meat or corned beef, bacon and sausage)	≥5.42 vs. ≤0.94 (times/week)	1.7 (1.3–2.2)	Age group, province, education, body mass index, sex, alcohol drinking (grams/day), pack-years smoking, total vegetable and fruit intake, and total energy intake
De stefani et al. (2012) Uruguay [56]	CC	274	2532	1996–2004	Processed meat (bacon, sausage, mortadella, salami, saucisson, hot dog, ham, and air-dried and salted lamb)	(Men) ≥28.3 vs. ≤11.4	1.93 (1.25–2.98) 4.51 (2.34–8.70)	Age, residence, body mass index, smoking status, smoking cessation, number of cigarettes smoked per day among current smokers, alcohol drinking, mate consumption, total energy, total vegetables and fruits, total white meat, and red meat intakes
						(Women) ≥ 28.3 vs. ≤11.4		
						(g/day)		
Wang et al. (2012) China [57]	CC	257	514	2008–2010	Red meat	>T3 vs. <T1 (tertiles)	1.3 (0.6–3.5)	Education, smoking, alcohol consumption, family history, total vegetable intake, total fruit intake, pickled food, soya products, total energy intake, and *H. pylori.*
Di maso et al. (2013) Italy and Switzerland [58]	CC	230	547	1991–2009	Red meat (beef, veal, pork, horsemeat, and half of the first course including meat sauce)	>90 vs. <60 (g/day)	1.38 (0.92–2.07)	Study center, age, sex, education, body mass index, tobacco smoking, alcohol drinking, vegetable consumption, and fruit consumption
Zamani et al. (2013) Iran [59]	CC	190	647	2004–2011	Red meat (fresh red meat and processed red meat) and white meat (poultry and fish)	>Q4 vs. <Q1 (quartiles)	1.87 (1.01–3.47)	Age, sex, energy intake, ethnicity, hot tea consumption, tooth brushing, cigarette smoking, SES, literacy, opium consumption, grains intake, dairy consumption, and vegetable and fruit intake.
							0.36 (0.19–0.68)	
Epplein et al. (2014) China [60]	CC	226	451	2002–2009	Red meat	>66.5 vs. ≤36.0 (g/day)	1.45 (0.93–2.28)	Age, smoking, history of gastritis, regular aspirin use, total energy intake, and high-risk *H. pylori* infection
Lin et al. (2014) China [61]	CC	107	209	2009–2010	Processed meat (salted meat)	>100 vs. never (g/week)	5.95 (1.33–25.62)	Age, gender, family history of cancer, ever smoking, alcohol drinking, fresh vegetable intake, fresh fruit intake, household income
Ellison-Loschmann et al. (2017) New Zealand [62]	CC	165	480	2009–2013	Red and white meat	Red meat (times/week)	0.59 (0.28–1.24)	Gender, age and weighted using post-stratification weights to account for differential non-response bias by deprivation quintile.
						≥5 vs. none		
						White meat (times/week)	0.54 (0.17–1.74)	
						≥5 vs. none		

CO: cohort study, CC: case-control study, ORs: odds ratios, RRs: relative risks, CI: confidence interval, MSI: microsatellite instability, GC: gastric cancer, BMI: body mass index, NSAID: Non-steroidal anti-inflammatory drugs, *H. pylori: helicobacter pylori*, SES: socioeconomic status.

**Table 2:** Stratified analysis (highest versus lowest categories) of red, processed, and white meat consumption and gastric cancer risk.

	Red Meat				Processed Meat				White Meat			
	No.	RR (95% CI)	Heterogeneity Test		No.	RR (95% CI)	Heterogeneity Test		No.	RR (95% CI)	Heterogeneity Test	
			*p*	I^2^%			*p*	I^2^%			*p*	I^2^%
Total	26	**1.41 (1.21–1.66)**	<0.001	69.6	33	**1.57 (1.37–1.81)**	<0.001	55.5	21	**0.80 (0.69–0.92)**	0.023	41.9
Study design												
Cohort studies	6	1.03 (0.83–1.28)	0.152	38.2	10	**1.24 (1.04–1.47)**	0.216	24.7	5	0.85 (0.63–1.16)	0.012	69.1
Case-control studies	20	**1.57 (1.30–1.89)**	<0.001	69.4	23	**1.79 (1.51–2.12)**	0.002	52.3	16	**0.77 (0.66–0.91)**	0.167	25.5
Population-based	12	**1.81 (1.41–2.33)**	0.045	51.3	11	**1.58 (1.32–1.89)**	0.28	17.2	9	**0.75 (0.61–0.93)**	0.006	62.5
Hospital-based	8	**1.42 (1.12–1.82)**	<0.001	71.2	12	**2.03 (1.55–2.68)**	0.001	64.7	7	0.81 (0.61–1.06)	0.324	13.9
Sex												
Men	4	1.09 (0.89–1.34)	0.42	0	5	**1.40 (1.13–1.74)**	0.388	3.3	3	0.81 (0.62–1.06)	0.287	19.8
Women	4	0.91 (0.73–1.12)	0.567	0	6	1.36 (0.84–2.18)	0.001	75	5	**0.67 (0.52–0.87)**	0.633	0
Geographic region												
Asia	9	**1.40 (1.04–1.89)**	0.002	67.9	9	**1.74 (1.22–2.48)**	0.033	52.2	9	**0.70 (0.57–0.85)**	0.335	11.9
Europe	8	**1.48 (1.15–1.92)**	0.008	63.2	10	**1.40 (1.14–1.73)**	0.038	49.3	4	0.79 (0.46–1.37)	0.005	76.9
North America	5	1.23 (0.92–1.65)	0.011	69.3	9	**1.36 (1.15–1.61)**	0.265	20.1	6	0.86 (0.73–1.01)	0.432	0
Latin America	3	**2.03 (1.01–4.06)**	0.004	81.8	5	**2.69 (1.76–4.12)**	0.023	64.9	1	0.98 (0.67–1.44)		
Oceania	1	0.59 (0.28–1.24)							1	0.54 (0.17–1.73)		
Anatomical subtype												
Cardia	6	1.19 (0.91–1.56)	0.128	41.5	5	0.95 (0.76–1.18)	0.52	0	5	1.12 (0.94–1.34)	0.776	0
Non-cardia	6	1.21 (0.89–1.63)	0.005	70.6	5	**1.34 (1.10–1.63)**	0.403	0.6	5	0.96 (0.75–1.24)	0.12	45.4
Histological subtype												
Intestinal (differentiated)	1	1.23 (0.61–2.51)			3	1.63 (0.87–3.04)	0.264	25	2	1.25 (0.77–2.03)	0.371	0
Diffuse (undifferentiated)	1	1.74 (0.93–3.24)			3	1.11 (0.44–2.82)	0.046	51.6	2	1.05 (0.32–3.43)	0.014	83.3
Quality score												
<7	3	1.39 (0.72–2.69)	0.004	81.6	5	**1.96 (1.55–2.49)**	0.341	11.4	2	0.80 (0.54–1.18)	0.941	0
≥7	23	**1.43 (1.21–1.68)**	<0.001	66	28	**1.50 (1.28–1.75)**	<0.001	56.8	19	**0.80 (0.68–0.93)**	0.011	47.7
Adjustments												
Total energy intake, yes	16	**1.37 (1.14–1.64)**	0.001	61.9	15	**1.47 (1.20–1.80)**	0.002	56.8	11	**0.80 (0.64–1.00)**	0.003	62.7
BMI, yes	11	**1.23 (1.01–1.50)**	0.004	61	7	**1.89 (1.51–2.36)**	0.105	42.9	7	0.86 (0.72–1.02)	0.299	17.2
Smoking, yes	15	**1.34 (1.11–1.61)**	<0.001	69.2	21	**1.68 (1.37–2.07)**	<0.001	65	11	0.84 (0.70–1.02)	0.008	56.7
Alcohol drinking, yes	14	**1.23 (1.01–1.49)**	<0.001	71.8	9	**2.34 (1.81–3.02)**	0.042	50.1	11	0.88 (0.73–1.06)	0.041	47.3
Vegetable intake, yes	12	**1.35 (1.10–1.66)**	<0.001	70.9	12	**2.02 (1.60–2.56)**	0.007	57.3	10	0.79 (0.61–1.02)	0.01	58.2
Fruit intake, yes	13	**1.43 (1.16–1.78)**	<0.001	74	15	**1.72 (1.42–2.10)**	0.001	62.2	8	0.83 (0.63–1.10)	0.013	60.8
Salt intake, yes	1	**3.40 (1.79–6.46)**			2	1.91 (0.69–5.24)	0.052	73.6	1	0.93 (0.39–2.20)		
Socioeconomic status, yes	21	**1.40 (1.17–1.68)**	<0.001	72	20	**1.57 (1.33–1.85)**	0.007	49.5	15	**0.80 (0.67–0.96)**	0.011	51.3
*Helicobacter pylori*, yes	3	**1.88 (1.04–3.40)**	0.075	61.5	1	1.14 (0.55–2.37)			1	0.93 (0.39–2.21)		

RR: relative risk, CI: confidence interval, BMI: body mass index, No.: number of studies, *p*: *p* value of Q test.

**Table 3:** Stratified dose–response analysis of red, processed, and white meat consumption and gastric cancer risk.

	Red Meat (per 100 g/day)				Processed Meat (per 50 g/day)				White Meat (per 100 g/day)			
	No.	RR (95% CI)	Heterogeneity Test		No.	RR (95% CI)	Heterogeneity Test		No.	RR (95% CI)	Heterogeneity Test	
			*p*	I^2^%			*p*	I^2^%			*p*	I^2^%
Total	18	**1.26 (1.11–1.42)**	<0.001	70.3	19	**1.72 (1.36–2.18)**	<0.001	72.1	14	0.86 (0.64–1.15)	0.01	52.8
Study design												
Cohort studies	4	1.08 (0.90–1.28)	0.447	0	7	**1.21 (1.04–1.41)**	0.427	0	4	0.91 (0.74–1.12)	0.279	21.9
Case-control studies	14	**1.31 (1.13–1.52)**	<0.001	75	12	**2.17 (1.36–2.18)**	<0.001	76.7	10	0.66 (0.35–1.25)	0.005	62
Population-based	9	**1.17 (1.00–1.37)**	0.002	68	7	**1.56 (1.25–1.93)**	0.347	10.8	5	0.70 (0.32–1.57)	0.008	70.7
Hospital-based	5	**1.64 (1.28–2.09)**	0.008	71.2	5	**5.33 (2.06–13.82)**	<0.001	89.7	5	0.56 (0.16–1.96)	0.052	57.5
Sex												
Men	3	1.06 (0.90–1.26)	0.365	0.7	3	**1.40 (1.01–1.93)**	0.682	0	2	0.10 (0.01–1.96)	0.04	76.2
Women	3	0.91 (0.75–1.10)	0.572	0	4	1.59 (0.71–3.56)	0.181	38.5	3	**0.07 (0.01–0.36)**	0.578	0
Geographic region												
Asia	5	1.50 (0.97–2.33)	<0.001	85	4	**10.17 (2.87–35.97)**	0.192	36.6	6	0.57 (0.19–1.66)	0.008	67.8
Europe	6	**1.32 (1.13–1.55)**	0.145	39.1	5	**1.50 (1.01–2.22)**	0.016	67.1	2	0.43 (0.04–4.65)	0.06	71.8
North America	4	1.14 (0.99–1.32)	0.244	27.9	7	**1.37 (1.11–1.69)**	0.292	18.1	5	0.86 (0.66–1.14)	0.135	43
Latin America	2	**1.48 (1.07–2.03)**	0.029	79.1	3	**2.24 (1.12–4.50)**	<0.001	88.9				
Oceania	1	0.65 (0.35–1.22)							1	1.46 (0.64–3.33)		
Anatomical subtype												
Cardia	4	1.19 (0.80–1.77)	0.043	63.2	3	0.99 (0.81–1.21)	0.791	0	4	1.16 (0.98–1.37)	0.793	0
Non-cardia	4	1.37 (0.90–2.09)	0.005	76.7	3	**1.18 (1.01–1.37)**	0.348	5.2	4	0.84 (0.64–1.11)	0.19	36.9
Histological subtype												
Intestinal (differentiated)	1	1.06 (0.58–1.96)			1	1.27 (0.93–1.75)			1	1.34 (0.48–3.39)		
Diffuse (undifferentiated)	1	1.28 (0.71–2.28)			1	1.04 (0.75–1.43)			1	1.63 (0.73–3.71)		
Quality score												
<7	3	1.33 (0.78–2.26)	0.005	81.1	2	**8.62 (3.35–22.16)**	0.994	0	2	0.39 (0.04–4.21)	0.385	0
≥7	15	**1.27 (1.12–1.44)**	<0.001	67.1	17	**1.57 (1.26–1.96)**	<0.001	68.4	12	0.86 (0.64–1.17)	0.006	58.2
Adjustments												
Total energy intake, yes	9	**1.22 (1.07–1.40)**	0.052	48.1	7	**1.46 (1.21–1.77)**	0.232	25.8	6	0.84 (0.60–1.18)	0.058	53.2
BMI, yes	6	**1.18 (1.06–1.31)**	0.485	0	3	**1.81 (1.15–2.83)**	0.01	78.5	4	0.89 (0.75–1.07)	0.341	10.4
Smoking, yes	11	**1.18 (1.05–1.33)**	0.001	67.6	13	**2.21 (1.54–3.17)**	<0.001	76	8	0.84 (0.58–1.21)	0.005	65.6
Alcohol drinking, yes	11	**1.19 (1.04–1.36)**	0.001	67.1	7	**3.28 (1.87–5.76)**	<0.001	84.8	10	0.80 (0.55–1.16)	0.005	61.8
Vegetable intake, yes	9	**1.28 (1.13–1.44)**	0.05	48.4	8	**2.79 (1.66–4.68)**	<0.001	83.3	9	0.91 (0.72–1.13)	0.183	29.5
Fruit intake, yes	10	**1.32 (1.15–1.52)**	0.006	61.1	9	**1.78 (1.29–2.46)**	<0.001	83.1	7	0.92 (0.80–1.07)	0.62	0
Salt intake, yes	1	**2.64 (1.61–4.34)**			1	**2.35 (1.29–4.29)**			1	1.14 (0.62–2.08)		
Socioeconomic status, yes	13	**1.26 (1.09–1.47)**	<0.001	73	15	**1.91 (1.43–2.55)**	<0.001	77.7	11	0.80 (0.57–1.11)	0.01	57
Helicobacter pylori, yes	2	**2.01 (1.16–3.50)**	0.132	55.9					1	1.14 (0.62–2.08)		

RR: relative risk, CI: confidence interval, BMI: body mass index, No.: number of studies, *p*: *p* value of Q test.

## Supplementary Materials

The following are available online at https://www.mdpi.com/2072-6643/11/4/826/s1, Table S1: PRISMA 2009 Checklist, Table S2: Search terms, Table S3: The quality of cohort studies (Newcastle-Ottawa Scale) included in the meta-analysis, Table S4: The quality of case-control studies (Newcastle-Ottawa Scale) included in the meta-analysis, Figure S1: Comparison of the adjusted relative risks and 95% confidence intervals of gastric cancer for the highest versus lowest groups of red meat consumption, Figure S2: Comparison of the adjusted relative risks and 95% confidence intervals of gastric cancer for the highest versus lowest groups of processed meat consumption, Figure S3: Comparison of the adjusted relative risks and 95% confidence intervals of gastric cancer for the highest versus lowest groups of white meat consumption, Figure S4: Begg’s funnel plot of studies for red meat consumption and gastric cancer risk. (a) Funnel plot of the highest versus lowest categories of red meat consumption and gastric cancer risk.; (b) Funnel plot of 100g/day increase in red meat consumption and gastric cancer risk, Figure S5: Begg’s funnel plot of studies for processed meat consumption and gastric cancer risk. (a) Funnel plot of the highest versus lowest categories of processed meat consumption and gastric cancer risk.; (b) Funnel plot of 100g/day increase in processed meat consumption and gastric cancer risk, Figure S6: Begg’s funnel plot of studies for white meat consumption and gastric cancer risk. (a) Funnel plot of the highest versus lowest categories of white meat consumption and gastric cancer risk.; (b) Funnel plot of 100g/day increase in white meat consumption and gastric cancer risk.
