# Supplementary material for: Effect of Red, Processed, and White Meat Consumption on the Risk of Gastric Cancer: An Overall and Dose–Response Meta-Analysis
Source: Nutrients. 2019 Apr 11;11(4):826. doi: 10.3390/nu11040826 (PMC6520977; doi:10.3390/nu11040826)
Supplement: Supplementary file 1 [file nutrients-11-00826-s001.pdf]

Supplementary Materials

Journal name: *Nutrients*

Title: Effect of red, processed, and white meat consumption on the risk of gastric cancer: An overall and dose-response meta-analysis

Authors: Seong Rae Kim, Kyuwoong Kim, Sang Ah Lee, Sung Ok Kwon, Jong-Koo Lee, NaNa Keum, Sang Min Park, MD, PhD.

Corresponding author: Sang Min Park, MD, PhD
Department of Family Medicine, Seoul National University Hospital, Seoul National University College of Medicine, 28 Yunkeon-dong, Jongro-gu, Seoul 03080, South Korea
Tel: +82-2-2072-3331 Fax: +82-2-766-3276
E-mail address: smpark.snuh@gmail.com

Table S1. PRISMA 2009 Checklist

Section/topic	#	Checklist item	Reported on page #
**TITLE**			
Title	1	Identify the report as a systematic review, meta-analysis, or both.	1
**ABSTRACT**			
Structured summary	2	Provide a structured summary including, as applicable: background; objectives; data sources; study eligibility criteria, participants, and interventions; study appraisal and synthesis methods; results; limitations; conclusions and implications of key findings; systematic review registration number.	1
**INTRODUCTION**			
Rationale	3	Describe the rationale for the review in the context of what is already known.	1-2
Objectives	4	Provide an explicit statement of questions being addressed with reference to participants, interventions, comparisons, outcomes, and study design (PICOS).	2
**METHODS**			
Protocol and registration	5	Indicate if a review protocol exists, if and where it can be accessed (e.g., Web address), and, if available, provide registration information including registration number.	NA
Eligibility criteria	6	Specify study characteristics (e.g., PICOS, length of follow-up) and report characteristics (e.g., years considered, language, publication status) used as criteria for eligibility, giving rationale.	2, 11-12
Information sources	7	Describe all information sources (e.g., databases with dates of coverage, contact with study authors to identify additional studies) in the search and date last searched.	2, 11-12
Search	8	Present full electronic search strategy for at least one database, including any limits used, such that it could be repeated.	2, 11-12
Study selection	9	State the process for selecting studies (i.e., screening, eligibility, included in systematic review, and, if applicable, included in the meta-analysis).	2, 11-12
Data collection process	10	Describe method of data extraction from reports (e.g., piloted forms, independently, in duplicate) and any processes for obtaining and confirming data from investigators.	2, 11-12

Data items	11	List and define all variables for which data were sought (e.g., PICOS, funding sources) and any assumptions and simplifications made.	2, 11-12
Risk of bias in individual studies	12	Describe methods used for assessing risk of bias of individual studies (including specification of whether this was done at the study or outcome level), and how this information is to be used in any data synthesis.	2, 11-12
Summary measures	13	State the principal summary measures (e.g., risk ratio, difference in means).	2, 11-12
Synthesis of results	14	Describe the methods of handling data and combining results of studies, if done, including measures of consistency (e.g., I^2) for each meta-analysis.	2, 11-12

**Section/topic**	**#**	**Checklist item**	**Reported on page #**
Risk of bias across studies	15	Specify any assessment of risk of bias that may affect the cumulative evidence (e.g., publication bias, selective reporting within studies).	2, 11-12
Additional analyses	16	Describe methods of additional analyses (e.g., sensitivity or subgroup analyses, meta-regression), if done, indicating which were pre-specified.	2, 11-12
**RESULTS**			
Study selection	17	Give numbers of studies screened, assessed for eligibility, and included in the review, with reasons for exclusions at each stage, ideally with a flow diagram.	11-24
Study characteristics	18	For each study, present characteristics for which data were extracted (e.g., study size, PICOS, follow-up period) and provide the citations.	11-24
Risk of bias within studies	19	Present data on risk of bias of each study and, if available, any outcome level assessment (see item 12).	11-24
Results of individual studies	20	For all outcomes considered (benefits or harms), present, for each study: (a) simple summary data for each intervention group (b) effect estimates and confidence intervals, ideally with a forest plot.	11-24
Synthesis of results	21	Present results of each meta-analysis done, including confidence intervals and measures of consistency.	11-24
Risk of bias across studies	22	Present results of any assessment of risk of bias across studies (see Item 15).	11-24
Additional analysis	23	Give results of additional analyses, if done (e.g., sensitivity or subgroup analyses, meta-regression [see Item 16]).	11-24
**DISCUSSION**			

Summary of evidence	24	Summarize the main findings including the strength of evidence for each main outcome; consider their relevance to key groups (e.g., healthcare providers, users, and policy makers).	24-26
Limitations	25	Discuss limitations at study and outcome level (e.g., risk of bias), and at review-level (e.g., incomplete retrieval of identified research, reporting bias).	24-26
Conclusions	26	Provide a general interpretation of the results in the context of other evidence, and implications for future research.	26
**FUNDING**			
Funding	27	Describe sources of funding for the systematic review and other support (e.g., supply of data); role of funders for the systematic review.	26

From: Moher D, Liberati A, Tetzlaff J, Altman DG, The PRISMA Group (2009). Preferred Reporting Items for Systematic Reviews and Meta-Analyses: The PRISMA Statement. PLoS Med 6(7): e1000097. doi:10.1371/journal.pmed1000097

For more information, visit: **www.prisma-statement.org**.

Table S2. Search terms

**MEDLINE (PubMed)**	(“meat”[tw] OR “meats”[tiab] OR "meat product"[tiab] OR "meat products"[tw] OR "red meat"[tw] OR "red meats"[tiab] OR beef[tiab] OR veal[tiab] OR goat[tiab] OR lamb[tiab] OR pork[tiab] OR mutton[tiab] OR sausage[tiab] OR sausages[tiab] OR ham[tiab] OR hams[tiab] OR pastrami[tiab] OR bacon[tiab] OR bacons[tiab] OR salami[tiab] OR salamis[tiab] OR "hot dog"[tiab] OR "hot dogs"[tiab] OR "animal food"[tiab] OR "animal foods"[tiab] OR "animal protein"[tiab] OR "animal proteins"[tiab] OR diet[tiab] OR diets[tiab] OR dietary[tiab] OR “white meat”[tiab] OR “poultry”[tiab] OR “chicken”[tiab] OR “duck”[tiab] OR “turkey”[tiab] OR “rabbit”[tiab]) AND (“gastric cancer”[tiab] OR “gastric neoplasm”[tiab] OR “stomach cancer”[tiab] OR “stomach neoplasm”[tiab] OR “gastric malignancy”[tiab] OR “stomach malignancy”[tiab] OR “gastric tumor”[tiab] OR “stomach tumor”[tiab])
**EMBASE**<br>**Cochrane Library**	(“meat” OR “meats” OR "meat product" OR "meat products" OR "red meat" OR "red meats" OR beef OR veal OR goat OR lamb OR pork OR mutton OR sausage OR sausages OR ham OR hams OR pastrami OR bacon OR bacons OR salami OR salamis OR "hot dog" OR "hot dogs" OR "animal food" OR "animal foods" OR "animal protein" OR "animal proteins" OR "diet" OR "diets" OR "dietary" OR “white meat” OR “poultry” OR “chicken” OR “duck” OR “turkey” OR “rabbit”) AND ("gastric cancer" OR "gastric neoplasm" OR "stomach cancer" OR "stomach neoplasm" OR "gastric malignancy" OR "stomach malignancy" OR "gastric tumor" OR "stomach tumor")

Table S3. The quality of cohort studies (Newcastle-Ottawa Scale) included in the meta-analysis [a]

First author, year	Representativeness of the exposed cohort	Selection of the unexposed cohort	Ascertainment of exposure	Outcome of interest not present at start of study	Control for important factor or additional factors [b]	Outcome assessment	Follow-up long enough for outcomes to occur [c]	Adequacy of follow-up of cohorts [d]	Total quality scores
Nomura A, 1990	☆	☆	☆	☆	☆☆	☆	☆	-	8
Zheng, 1995	☆	☆	☆	☆	☆☆	☆	☆	☆	9
Galanis D J, 1998	☆	☆	☆	☆	☆	☆	☆	☆	8
Knekt P, 1999	☆	☆	☆	☆	☆☆	☆	☆	☆	9
Huang, 2000	☆	☆	☆	☆	☆☆	☆	☆	☆	9
González CA, 2006	☆	☆	-	☆	☆☆	☆	☆	☆	8
Larsson SC, 2006	☆	☆	☆	☆	☆☆	☆	☆	☆	9
Cross AJ, 2011	☆	☆	☆	☆	☆☆	☆	☆	☆	9
Daniel	☆	☆	☆	☆	☆☆	☆	☆	☆	9
Keszei AP, 2012	☆	☆	☆	☆	☆☆	☆	☆	☆	9

Wie, 2014	☆	☆	☆	☆	☆☆	☆	-	☆	8

a. A maximum of one star was assigned for almost all of the items.

b. A maximum of two stars was assigned for this item. Studies that adjusted for age received one star, whereas studies that adjusted for other important confounders additionally such as family history of cancer or helicobacter pylori infection or health behaviors (such as smoking or alcohol drinking) received an additional star.

c. A cohort study with a follow-up period >8 y was assigned one star.

d. A cohort study with a follow-up rate >75% was assigned one star.

Table S4. The quality of case-control studies (Newcastle-Ottawa Scale) included in the meta-analysis [a]

First author, year	Adequate definition of cases	Representativeness of cases	Selection of controls	Definition of controls	Control for important factor or additional factors [b]	Exposure assessment	Same method of ascertainment for all subjects	Nonresponse rate [c]	Total quality scores
Lee HH, 1990	☆	☆	-	-	☆	-	☆	-	4
Boeing H, 1991	-	☆	-	☆	☆☆	-	☆	-	5
González CA, 1991	☆	☆	-	☆	☆	☆	☆	-	6
Hoshiyama Y, 1992	☆	☆	☆	☆	☆☆	☆	☆	-	8
Muñoz SE, 1997	☆	☆	-	☆	☆	☆	☆	-	6
Ward MH, 1997	☆	☆	☆	☆	☆	☆	☆	-	7
Ji BT, 1998	☆	☆	☆	-	☆☆	☆	☆	-	7
Ward MH, 1999	☆	☆	☆	-	☆☆	☆	☆	-	7
Tavani A, 2000	☆	☆	-	☆	☆☆	☆	☆	-	7
Takezaki T,	☆	☆	☆	-	☆☆	☆	☆	-	7

2001									
Palli D, 2001	☆	☆	☆	-	☆☆	☆	☆	-	7
Kim HJ, 2002	☆	☆	-	☆	☆☆	☆	☆	-	7
Ito LS, 2003	☆	☆	-	☆	☆☆	☆	☆	-	7
Nomura AM, 2003	☆	☆	☆	-	☆☆	☆	☆	-	7
Lissowska J, 2004	☆	☆	☆	-	☆☆	☆	☆	-	7
De Stefani E, 2004	☆	☆	-	☆	☆☆	☆	☆	-	7
Phukan RK, 2006	☆	☆	-	☆	☆	☆	☆	-	6
Strumylaitė L, 2006	☆	☆	-	-	☆	☆	☆	-	5
Wu AH, 2007, USA	☆	☆	☆	☆	☆☆	☆	☆	☆	9
Navarro Silvera SA, 2008	☆	☆	☆	-	☆☆	☆	☆	-	7
Hu JF, 2008	☆	☆	☆	-	☆☆	☆	☆	-	7

Aune D, 2009	☆	☆	-	☆	☆☆	☆	☆	-	7
Aune D, 2009	☆	☆	-	☆	☆☆	☆	☆	-	7
Pourfarzi E, 2009	☆	☆	☆	☆	☆☆	☆	☆	-	8
Gao Y, 2011, China	☆	☆	-	☆	☆	☆	☆	☆	7
Hu, 2011	☆	☆	☆	☆	☆☆	☆	☆	-	8
De Stefani, 2012	☆	☆	-	☆	☆☆	☆	☆	☆	8
Wang, 2012	☆	☆	☆	☆	☆	☆	☆	-	7
Di maso, 2013	☆	☆	☆	-	☆☆	-	☆	-	6
Zamani, 2013	☆	☆	☆	☆	☆☆	☆	☆	-	8
Epplein, 2014	☆	☆	☆	☆	☆☆	☆	☆	-	8
Lin, 2014	☆	☆	-	☆	☆☆	☆	☆	-	7
Ellison-Loschmann, 2017	☆	☆	☆	-	☆☆	☆	☆	-	7

a. A maximum of one star was assigned for almost all of the items.

b. A maximum of two stars was assigned for this item. Studies that adjusted for age received one star, whereas studies that adjusted for other important confounders additionally such as family history

of cancer or helicobacter pylori infection or health behaviors (such as smoking or alcohol drinking) received an additional star.

c. A maximum of one star was assigned for this item if there was no significant difference in the response rate between cases and controls by using the chi-square test ($P>0.05$)

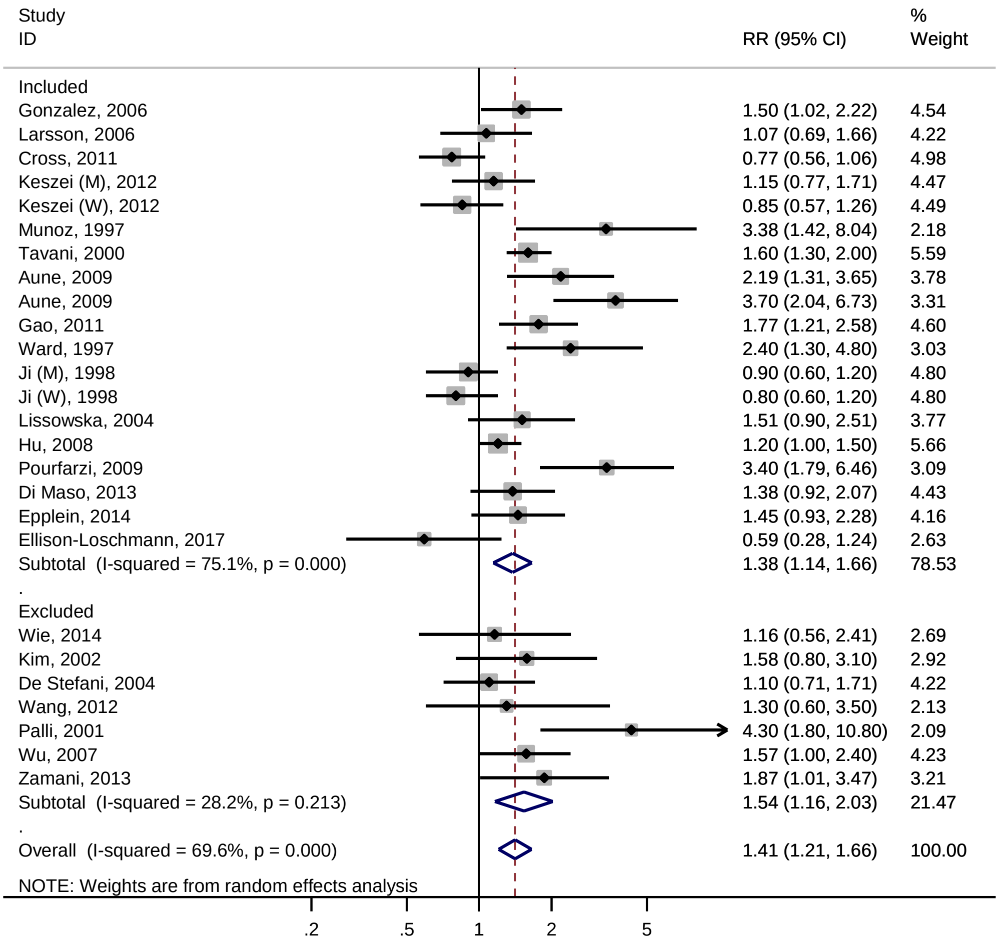

Figure S1. Comparison of the adjusted relative risks and 95% confidence intervals of gastric cancer for the highest versus lowest groups of red meat consumption (studies included for the dose-response analysis versus studies not included for the dose-response analysis). Squares mean each study's relative risks. Horizontal lines mean 95% confidence intervals. Diamonds mean the summary relative risks and 95% confidence intervals.

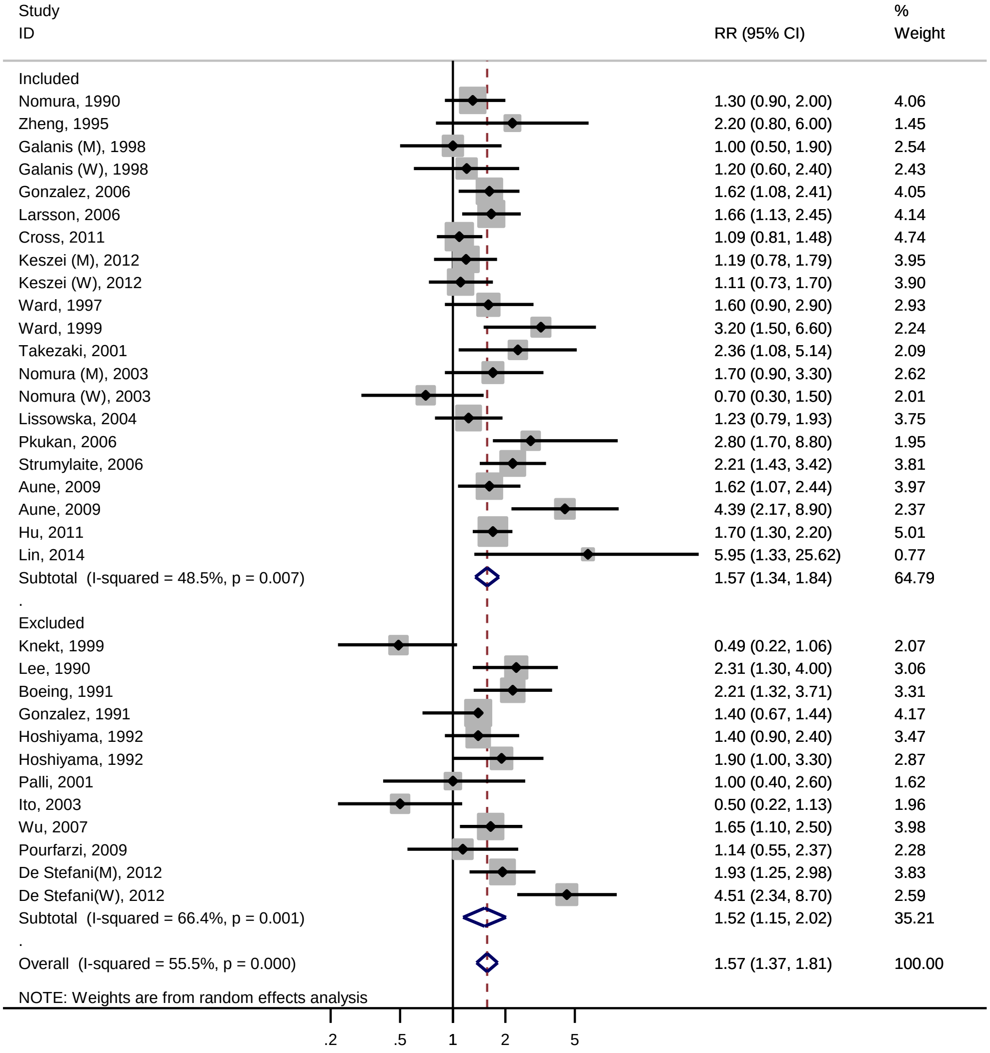

Figure S2. Comparison of the adjusted relative risks and 95% confidence intervals of gastric cancer for the highest versus lowest groups of processed meat consumption (studies included for the dose-response analysis versus studies not included for the dose-response analysis). Squares mean each study's relative risks. Horizontal lines mean 95% confidence intervals. Diamonds mean the summary relative risks and 95% confidence intervals.

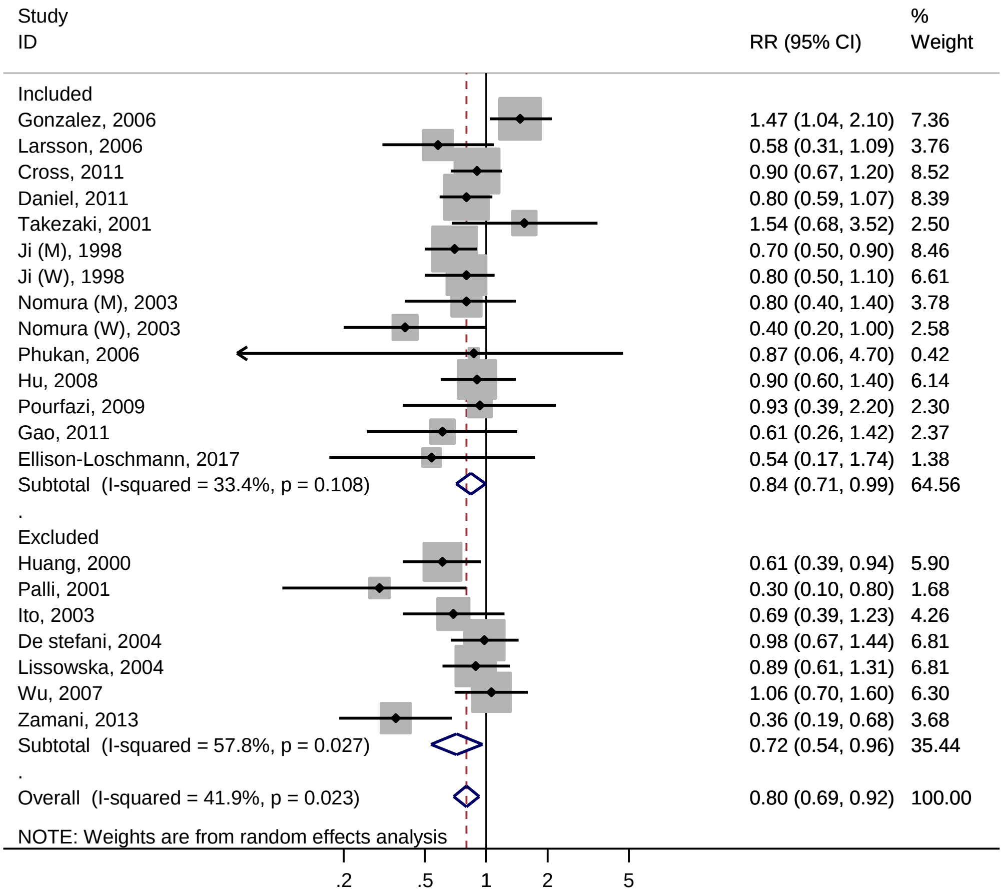

Figure S3. Comparison of the adjusted relative risks and 95% confidence intervals of gastric cancer for the highest versus lowest groups of white meat consumption (studies included for the dose-response analysis versus studies not included for the dose-response analysis). Squares mean each study's relative risks. Horizontal lines mean 95% confidence intervals. Diamonds mean the summary relative risks and 95% confidence intervals.

a. Highest versus lowest categories

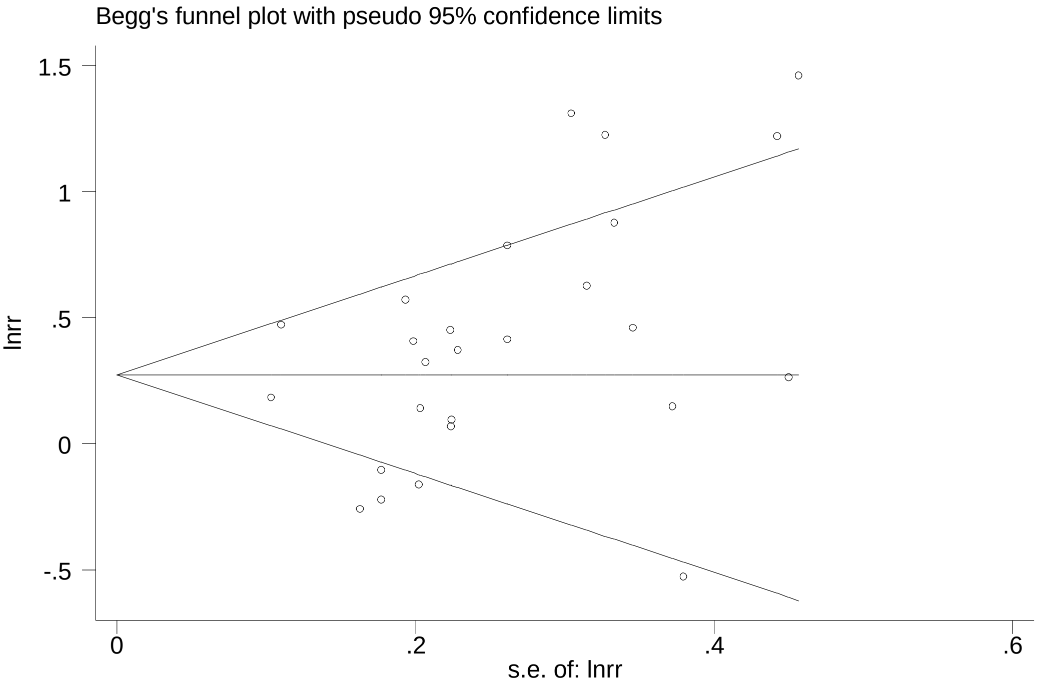

b. Red meat (per 100g/day)

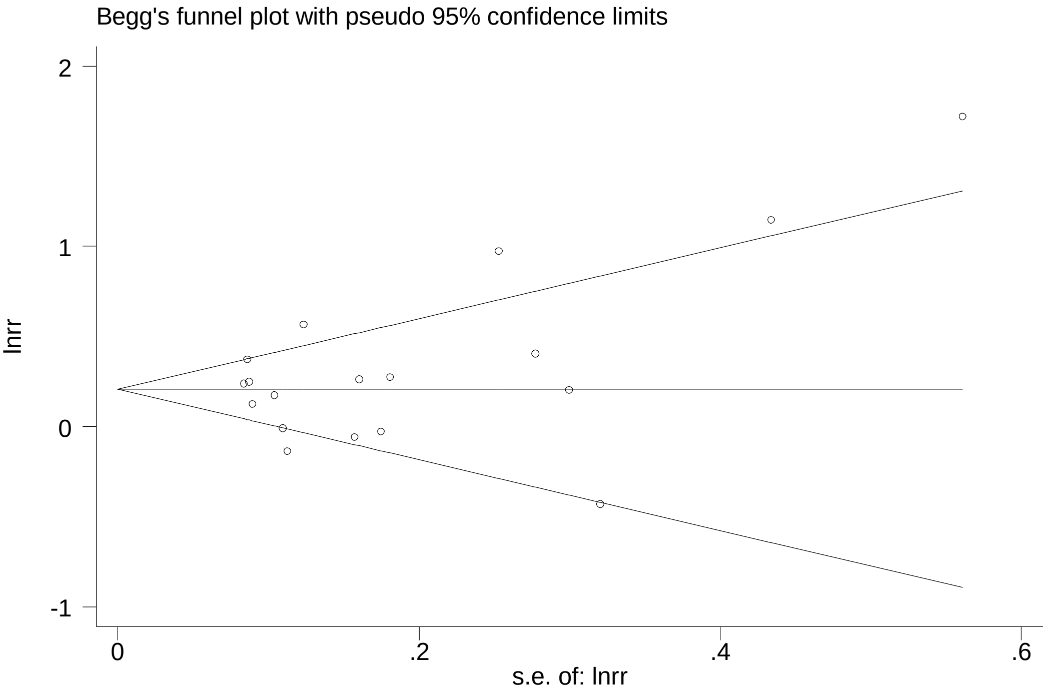

Figure S4. Begg's funnel plot of studies for red meat consumption and gastric cancer risk. (a) Funnel plot of the highest versus lowest categories of red meat consumption and gastric cancer risk.; (b) Funnel plot of 100g/day increase in red meat consumption and gastric cancer risk.

a. Highest versus lowest categories

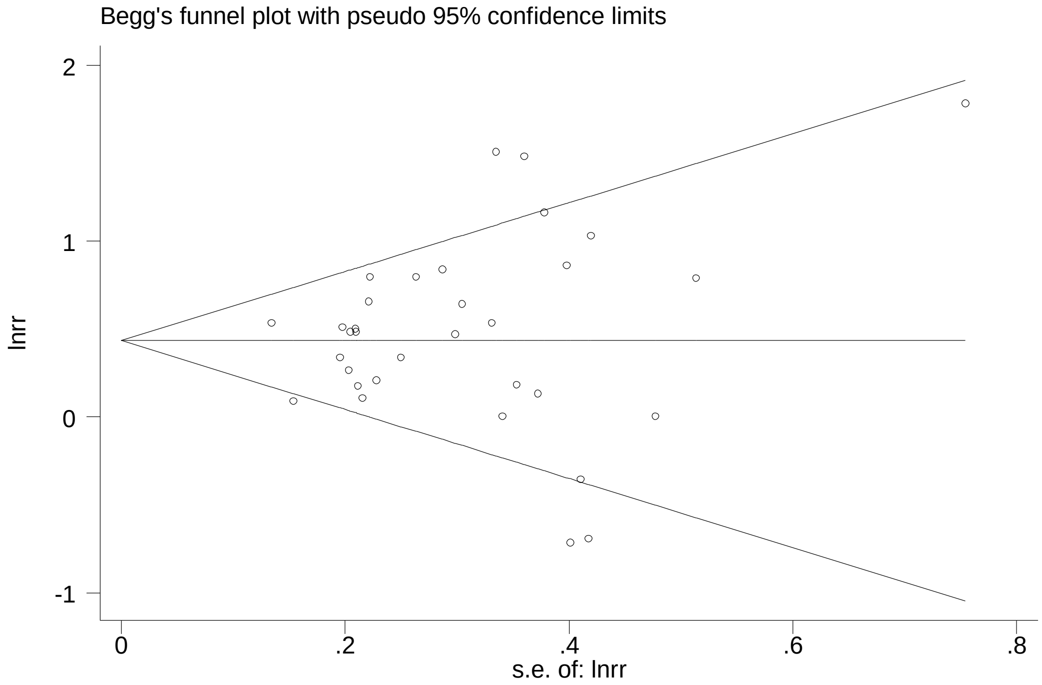

b. Processed meat (per 50g/day)

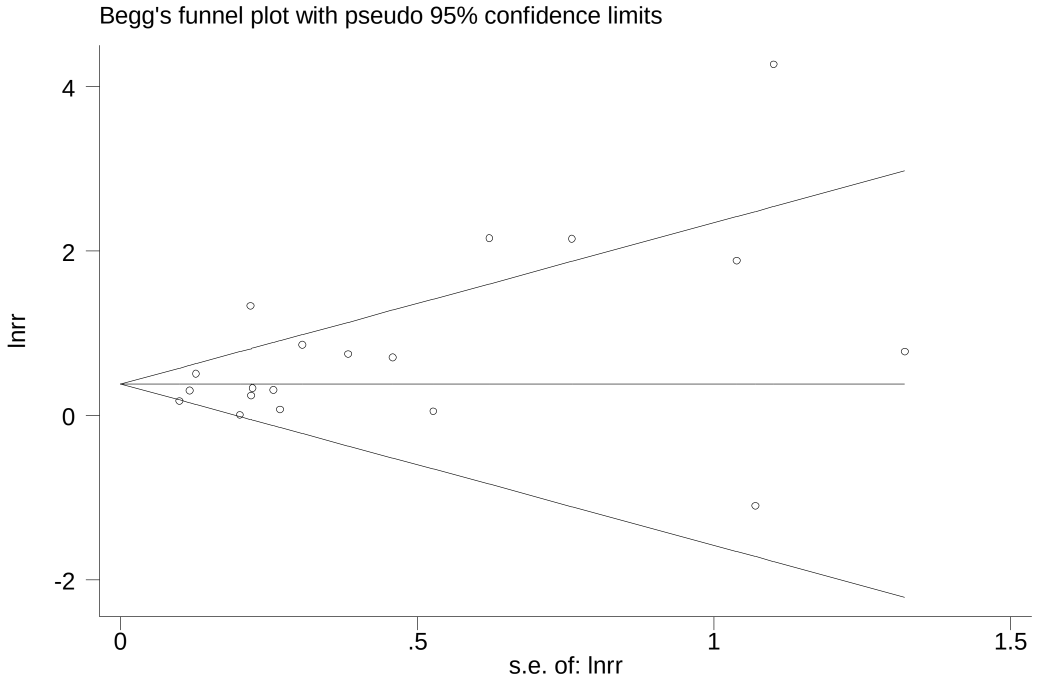

Figure S5. Begg's funnel plot of studies for processed meat consumption and gastric cancer risk. (a) Funnel plot of the highest versus lowest categories of processed meat consumption and gastric cancer risk.; (b) Funnel plot of 100g/day increase in processed meat consumption and gastric cancer risk.

a. Highest versus lowest categories

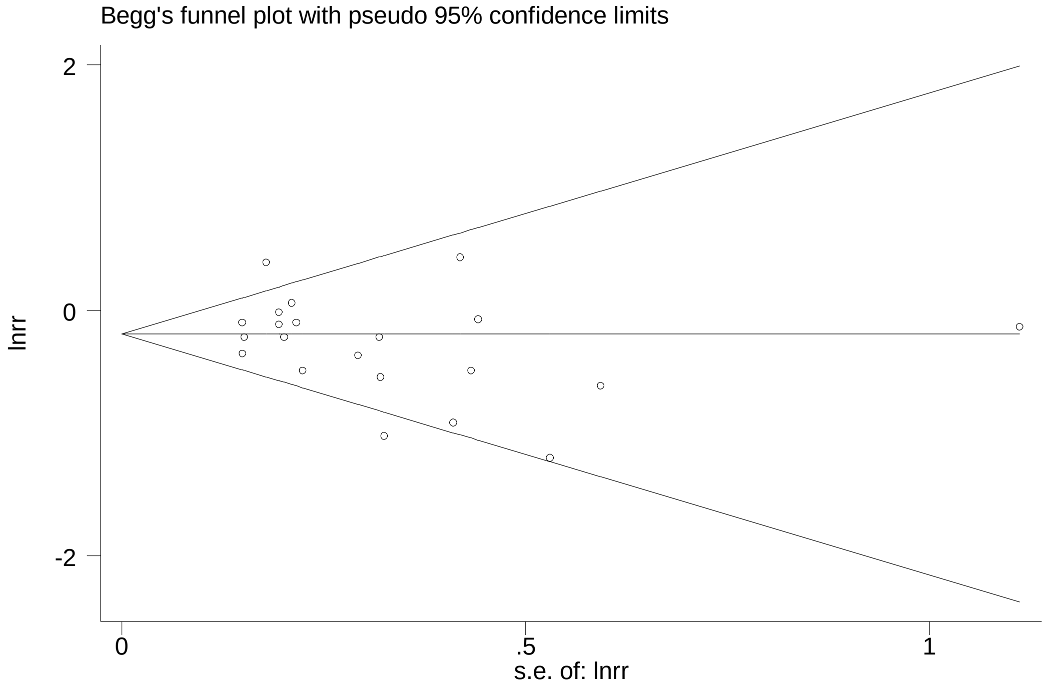

b. White meat (per 100g/day)

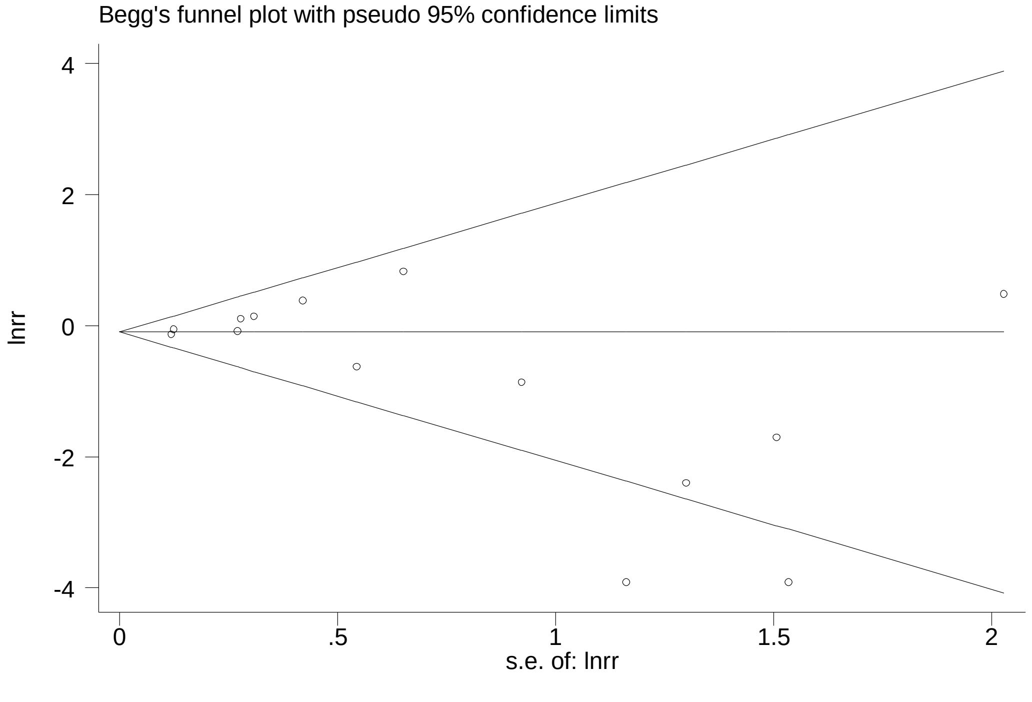

Figure S6. Begg's funnel plot of studies for white meat consumption and gastric cancer risk. (a) Funnel plot of the highest versus lowest categories of white meat consumption and gastric cancer risk.; (b) Funnel plot of 100g/day increase in white meat consumption and gastric cancer risk.
